# Meclofenamic acid selectively inhibits FTO demethylation of m^6^A over ALKBH5

**DOI:** 10.1093/nar/gku1276

**Published:** 2014-12-01

**Authors:** Yue Huang, Jingli Yan, Qi Li, Jiafei Li, Shouzhe Gong, Hu Zhou, Jianhua Gan, Hualiang Jiang, Gui-Fang Jia, Cheng Luo, Cai-Guang Yang

**Affiliations:** 1 CAS Key Laboratory of Receptor Research, Shanghai Institute of Materia Medica, Chinese Academy of Sciences, Shanghai 201203, China; 2 Synthetic and Functional Biomolecules Center, Beijing National Laboratory for Molecular Sciences, Key Laboratory of Bioorganic Chemistry and Molecular Engineering of Ministry of Education, College of Chemistry and Molecular Engineering, Peking University, Beijing 100871, China; 3 School of Life Sciences, Fudan University, Shanghai 200433, China; 4 State Key Laboratory of Drug Research, Shanghai Institute of Materia Medica, Chinese Academy of Sciences, Shanghai 201203, China

## Abstract

Two human demethylases, the fat mass and obesity-associated (FTO) enzyme and ALKBH5, oxidatively demethylate abundant *N*^6^-methyladenosine (m^6^A) residues in mRNA. Achieving a method for selective inhibition of FTO over ALKBH5 remains a challenge, however. Here, we have identified meclofenamic acid (MA) as a highly selective inhibitor of FTO. MA is a non-steroidal, anti-inflammatory drug that mechanistic studies indicate competes with FTO binding for the m^6^A-containing nucleic acid. The structure of FTO/MA has revealed much about the inhibitory function of FTO. Our newfound understanding, revealed herein, of the part of the nucleotide recognition lid (NRL) in FTO, for example, has helped elucidate the principles behind the selectivity of FTO over ALKBH5. Treatment of HeLa cells with the ethyl ester form of MA (MA2) has led to elevated levels of m^6^A modification in mRNA. Our collective results highlight the development of functional probes of the FTO enzyme that will (i) enable future biological studies and (ii) pave the way for the rational design of potent and specific inhibitors of FTO for use in medicine.

## INTRODUCTION

The methylation of internal adenosines at the *N*^6^ position (m^6^A) in messenger RNA (mRNA) was first observed several decades ago (1,2). A grasp of its regulatory function was missing, however, until the discovery of the fat mass and obesity-associated (FTO) enzyme in 2011. An m^6^A demethylase, FTO, was shown to regulate cellular levels of endogenous m^6^A residues, which strongly suggested that the m^6^A modification in mRNA is reversible and might be subject to dynamic regulation (3). The existence of the m^6^A demethylase FTO and the demonstration of the dynamic regulation of the levels of m^6^A have stimulated explorations of m^6^A modifications and related enzymes. Researchers have uncovered a wealth of unequivocal evidence for m^6^A function. The identification and characterization of the m^6^A methyltransferase tricomplex (METTL3–METTL14–WTAP) further highlights the biological significance of m^6^A methylation (4,5). The physiological relevance of this modification remains unclear, however. Two recent RNA ‘methylome’ studies have provided a map of m^6^A-modified mRNAs (6,7). Both found that the ubiquitous m^6^A modification plays a fundamental regulatory role in gene expression. In addition, binding proteins selectively recognize the dynamic m^6^A modification, research shows, in order to regulate translation status and the lifetime of mRNA (8). High-resolution mapping has revealed that methylation of adenosine to m^6^A in mRNA is particularly important for yeast meiosis (9). These studies have provided new insights into the distribution and functional role of m^6^A in mRNA (10,11).

FTO belongs to the family of Fe^2+^ and 2-oxoglutarate (2OG) dependent AlkB dioxygenases (12) and contributes to non-syndromic human obesity (13). Besides the internal m^6^A substrates in mRNA, FTO also oxidatively demethylates *N*^3^-methylthymine (dm^3^T) in single-stranded (ss) DNA and *N*^3^-methyluridine (m^3^U) in ssRNA *in vitro* (14), but has relatively lower activities compared to other AlkB family enzymes that catalyze a wide range of oxidative reactions (15–19). The FTO gene was initially shown to influence human obesity and energy utilization (20,21). In addition, reports indicate the involvement of the FTO protein itself in various diseases (22–26). Such discoveries make FTO an increasingly interesting target with respect to its functional links to human diseases. Another member of the AlkB family, ALKBH5, was also identified as an m^6^A demethylase of mRNA using Fe^2+^ and cofactor 2OG, which together function to oxidatively remove the methyl group in m^6^A-containing substrates (27). Both FTO and ALKBH5 are localized to nuclei and colocalize with nuclear speckles, indicating the effects of methylation on splicing. Indeed, knockdown of the ALKBH5 gene was shown to affect splicing in tissue culture cells, and displayed a sterility phenotype in male mice. The discovery of these two m^6^A demethylases within the scientific community highlights the importance of m^6^A modification in basic biology and human disease. Studies that focus on the inhibition of m^6^A demethylation will likely (i) shed light on the science of ‘RNA epigenetics’ in chemical biology and (ii) hold promise for future therapeutic developments (28,29).

The functions and mechanistic studies of *Escherichia coli* AlkB (30–33), and its human homologs, ALKBH1-8 (34–36), greatly facilitate the development of inhibitors targeting m^6^A demethylases. Of particular note is a strategy that involves a 2OG-tethering strategy of simultaneously occupying both the 2OG- and substrate-binding sites. The practice of linking 2OG derivatives with the substrate analogs has been successfully applied to the development of selective inhibitors of histone demethylases containing a jumonji domain (37–39). Later on, researchers have applied a similar strategy in order to develop the inhibitors for the AlkB enzyme with success (40). Interestingly, some of the inhibitors have been shown to be selective over other 2OG oxygenases *in vitro*. The major concern here, however, is that these inhibitors are derivatives of 2OG, and therefore cellular 2OG might compete with them. We and others are working concurrently on the development of FTO inhibitors (41). Our initial steps have led to the identification of the natural product rhein as the first inhibitor of FTO through structure-based virtual screening (42). Rhein, unlike the generic inhibitors of either chemical mimics of 2OG or chelators of iron (43), competitively disrupts FTO from binding to the m^6^A substrate, and promotes the thermal stability of FTO by directly binding. Rhein also actively increases cellular levels of m^6^A in mRNA. The structural complex shows that rhein does indeed bind to the nucleic acid-binding site (44). However, rhein shows little selectivity for the AlkB subfamily, which eclipses its utility as a specific functional probe of FTO inside the cell. The 2OG-tethering strategy was also used to develop the cell active FTO inhibitors; during the revision of this work, these inhibitors proved highly selective *in vitro* for the AlkB subfamily. *In vivo* selectivity remains unclear, however (45).

In order to avoid competition with internal 2OG, we employed an alternative approach to the identification of selective inhibitors of FTO. We performed a high-throughput fluorescence polarization (FP) assay to compare the differences in the displacement of m^6^A-containing ssDNA binding to FTO and ALKBH5, respectively, in the presence of compounds. This screening led directly to the discovery of meclofenamic acid (MA) that specifically inhibits FTO over ALKBH5. Herein, we focus on a mechanistic study of the selective inhibition of m^6^A demethylase. Our results will create opportunities for understanding the development of specific functional probes that may target FTO for biological and therapeutic purposes.

## MATERIALS AND METHODS

### Protein expression and purification

The expression and purification of FTO_ΔN31_ (encoding for His-tag human FTO with N-terminal 31 residues truncated) was modified from previously reported methods (14). *E. coli* BL21(DE3) cells transformed with the pET28a-*FTO_ΔN31_* plasmids were grown at 37ºC to *A*_*600*_ 0.6–0.8 and induced by 0.5 mM Isopropyl β-D-1-thiogalactopyranoside at 16ºC for 16 h. The cell pellets were harvested and stored at −80ºC. The cells were resuspended and sonicated in 20 mM Tris-HCl, pH 8.0, 300 mM NaCl in the presence of 5% glycerol. The lysate was centrifuged and the supernatant was loaded onto a 5 ml HisTrap^TM^ HP column (GE Healthcare). The column was allowed to reach equilibrium with binding buffer (20 mM Tris-HCl, pH 8.0, 300 mM NaCl, 10 mM imidazole) and eluted with elution buffer (20 mM Tris-HCl, pH 8.0, 300 mM NaCl, 200 mM imidazole). The fractions were diluted and applied onto a 1 ml MonoQ column, and eluted with a linear gradient of 0–500 mM NaCl, followed by a gel filtration (Superdex 200) in 50 mM Tris-HCl, pH 8.0, 150 mM NaCl. The combined protein fractions were collected and concentrated to 20 mg/ml for storage.

The human *ALKBH5_66–292_* gene was cloned into the pET28a vector, encoding an N-terminal His-tagged protein. The protein was purified by affinity chromatography as described (46) and eluted with 500 mM imidazole in 20 mM Tris-HCl, pH 8.0 and 500 mM NaCl. The fractions were loaded on 12% sodium dodecyl sulphate-polyacrylamide gel electrophoresis (SDS-PAGE) for purity analysis. Finally, high purity of ALKBH5 protein was obtained for further bioassays.

### PAGE-based assay of the inhibition of m^6^A demethylation in ssDNA

The known PAGE-based procedures were performed in order to evaluate the inhibitory activities (14,42). FTO_ΔN31_ and ALKBH5_66–292_ proteins were purified as described above. The methylated 49 nt ssDNA substrate sequence covered a DpnII cleavage site [5′-TAGACATTGCCATTCTCGATAGG(dm^6^A)TCCGGTCAAACCTAGACGAATTCCA-3′]. The reaction mixtures contained 50 mM Tris-HCl, pH 7.5, 1 μM ssDNA, 1 μM FTO or 3 μM ALKBH5, 300 μM 2OG, 280 μM (NH_4_)_2_Fe(SO_4_)_2_, 2 mM L-ascorbic acid, and compounds at varying concentrations. After incubation at room temperature for 2 h, the reactions were heated to quench. The ssDNA was annealed to the complementary strand for DpnII digestion. The digestion samples were checked on 15% non-reducing PAGE, with Gel-Red staining to measure the intensity of bands.

### High performance liquid chromatography (HPLC)-based assay of the inhibition of m^6^A demethylation in ssDNA or ssRNA

Based on the published HPLC-based protocol (3,27), reactions were set up to quantitatively verify MA inhibition of FTO and ALKBH5 demethylation activities. The reactions contained 1 μM FTO or 5 μM ALKBH5, 5 μM 20 nt ssDNA (5′-CTCGATACG (dm^6^A)TCCGGTCAAA-3′) or 5 μM 20 nt ssRNA (5′-CUCGAUACG (m^6^A)UCCGGUCAAA-3′) in 50 mM Tris-HCl, pH 7.5, 300 μM 2OG, 280 μM (NH_4_)_2_Fe(SO_4_)_2_, 2 mM L-ascorbic acid, and serial concentrations of compounds, incubated at 25ºC for 2 h for ssDNA or 30 min for ssRNA. The reactions were terminated by heating and the mixtures were digested by nuclease P1 and alkaline phosphatase. The digestion products were separated and analyzed on an HPLC system with a Phenomenex Luna 5μ C18 analyses column (150 × 4.6 mm). The mobile phase binding buffer (25 mM NaH_2_PO_4_) and elution buffer (acetonitrile) were mixed and flown at a rate of 1 ml/min. The detection wavelength was 266 nm. To determine the IC_50_, different concentrations (0.5, 1, 5, 10, 50, 100, 500 and 1000 μM) of inhibitor MA were used to calculate the inhibitory percentages of demethylation, using nonlinear regression, dose-response fit on GraphPad Prism 5.0^TM^. All reactions were performed in triplicate.

### Differential scanning fluorimetry

A 30 μl mixture containing 2 μM FTO or ALKBH5 protein, 5 × SYPRO Orange dye (Invitrogen, commercial stock is 5000×) and tested compounds (or 1.5% dimethyl sulfoxide (DMSO) control) was set up for differential scanning fluorimetry (47). Assays were performed using real-time polymerase chain reaction detection system (ABI 7500 Fast) and heated from 25ºC to 95ºC at 1% ramp rate. The excitation and emission wavelength is 492 nm and 610 nm, respectively. Melting curves were analyzed with Graphpad Prism 5.0^TM^. All reactions were performed in triplicate.

### FP assay

The fluorescein-labeled ssDNA substrate [5′-ATTGTCA (dm^6^A) CAGCAGA-FAM-3′] was used in the FP assay. Serial dilution compounds were added to a reaction mixture containing 50 mM borate buffer, pH 7.5, 3 μM FTO and 20 nM ssDNA or 125 nM ALKBH5 and 25 nM ssDNA, respectively, to a final volume of 100 μl. After incubation at room temperature for 30 min, FP was measured on a Microplate Reader at the wavelength of 480 nm for excitation and 520 nm for emission, respectively. The inhibitory curves and parameters were calculated from nonlinear regression using GraphPad Prism 5.0^TM^.

### Crystallization and structure determination of FTO/MA complex

Crystals of FTO_ΔN31_ in complex with inhibitor MA were grown in 289 K using the vapor diffusion method. FTO_ΔN31_ proteins were incubated with 1 mM MnCl_2_, 3 mM *N*-Oxalylglycine (NOG) and 1.5 mM MA on ice for 30 min and mixed with a reservoir solution of 100 mM sodium citrate, pH 5.6, 11.5% (w/v) polyethylene glycol (PEG) 3350, and 8% isopropanol at a volume ratio of 1:2. The crystals were cryoprotected using 20% (v/v) glycerol and then flash-frozen in liquid nitrogen. Diffraction data were collected at Shanghai Synchrotron Radiation Facility beamline 17U. All X-ray data were processed using HKL2000 programs (48) and converted to structure factors within the CCP4 program (49). The structure was solved by molecular replacement in Phaser using the structure of FTO/dm^3^T complex (PDB code 3LFM) as the searching model (50). The model of structural complex FTO/MA was manually built using COOT (51) and computational refinement was carried out with the program REFMAC5 (52) in the CCP4 suite.

### Cell line and antibodies

Human HeLa cells were routinely grown in a humidified incubator at 37°C with 5% CO_2_ in Dulbecco's modified Eagle's medium (Gibco) supplemented with 10% Fetal Bovine Serum (FBS) and 1% penicillin/streptomycin. HeLa cells were purchased from Cell Culture Center of Institute of Basic Medical Sciences (Chinese Academy of Medical Sciences, Beijing, China). The primary antibodies were purchased from commercial sources: Rabbit anti-FTO (5325-1; Epitomics), HRP-goat anti rabbit IgG (E030120-01; Earthox), HRP-goat anti mouse IgG (E030110-01; Earthox). Rabbit anti-ALKBH5 was gifted from Professor Yungui Yang (Beijing Institute of Genomics, Chinese Academy of Sciences).

### Analysis of MA2 conversion to MA inside cells

The Prominence LC-20A HPLC system coupled with an Applied Biosystems 5500 QTrap mass spectrometer operated in positive form and negative ion mode were used in order to analyze the MA2 intracellular metabolic product. A supposed product is MA, the acid form of MA2. MA's precursor ion and product ion m/z were 296.3 and 215.9, respectively. HeLa cells were harvested after exposure in the mediums with 80 μM MA2 for 24 h and washed thrice with phosphate buffered saline buffer. The cells were then diluted to 100 μl with water containing 0.1% formic acid (v/v), followed by the addition of 400 μl of cold methanol, vortexed sharply and combined with several shock freeze–thaw cycles. Samples were centrifuged and the supernatant was collected and dried using a vacuum evaporator. The residue was redissolved in 100 μl methanol and centrifuged at 10 000 × g at 4°C for 20 min in order to obtain a clear extract for liquid chromatography coupled to tandem mass spectrometry (LC-MS/MS) analysis.

### Inhibition of m^6^A demethylation in HeLa cells

The tested compounds were freshly dissolved into cell media before feeding to HeLa cells at varying concentrations as indicated. After a 24 h incubation period, cells were harvested and the total cellular RNA was isolated with TRIZOL Reagent (Invitrogen). mRNA was extracted using PolyATtract^®^ mRNA Isolation Systems (Promega), followed by further removing of contaminated rRNA using RiboMinus Transcriptome Isolation Kit (Invitrogen). The isolated mRNA samples were digested by nuclease P1 and alkaline phosphatase following standard protocol. The nucleosides were separated by reverse phase ultra-performance liquid chromatography coupled with online triple-quadrupole LC mass spectrometer detection, and quantified by comparison with the standard curve obtained from nucleoside standards running the same batch of samples. The ratio of m^6^A/A or m^6^A/U was calculated.

### RNA knockdown and overexpression of FTO and ALKBH5 by transient transfection

The target FTO mRNA sequence is 5′-AAAUAGCCGCUGCUUGUGAGA-3′. The FTO knockdown control (called Si control) used a non-sense siRNA with the sequence 5′-CAGGGTATCGACGATTACAAA-3′. The FTO plasmid used for overexpression was constructed full-length into mammalian vector pcDNA3 with N-terminal FLAG-tag. The FTO overexpression control (called Oe control) was transfected with an empty vector, pcDNA3. For the *in vivo* selectivity experiments, all the HeLa cells used were transfected with FTO SiRNA. ALKBH5 was overexpressed in the transfected HeLa cells with FTO siRNA using a mammalian expression vector. SiFTO Oe control was transfected with an empty vector of pEGFP. SiFTO Oe ALKBH5 was transfected with an ALKBH5 vector in full-length in siFTO cells. All transfections were performed using Lipofectamine RNAiMAX (Invitrogen) for siRNA and Lipofectamine 2000 (Invitrogen) for plasmid transfection following the manufacturer's protocols.

## RESULTS

### MA inhibits FTO demethylation in single-stranded nucleic acids *in vitro*

**Figure 1.**
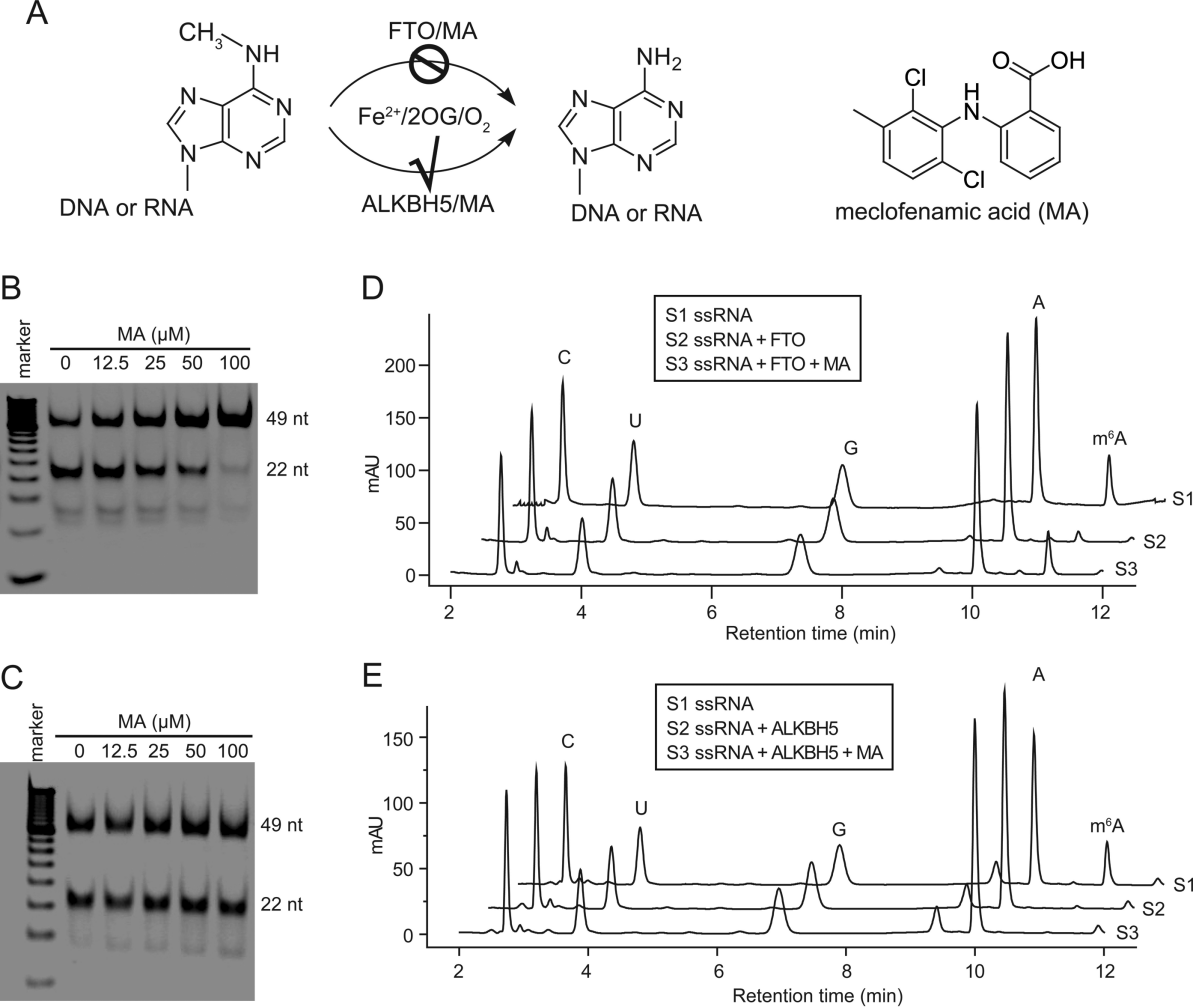
Selective inhibition of m^6^A demethylases *in vitro*. (**A**) Scheme of the reverse of m^6^A modification in single-stranded nucleic acids by FTO and ALKBH5, respectively. The chemical structure of MA is shown. Our research posed the following question: could the inhibition of FTO be selective and, if so, how? (**B**) Detection of inhibition of FTO demethylation on ssDNA using the restriction enzyme digestion assay. In PAGE image, the upper band is 49 nt DNA with dm^6^A incorporation, and the lower band represents the demethylated products after DpnII digestion. MA inhibits FTO demethylation in a dose-response manner. (**C**) Detection of inhibition of ALKBH5 demethylation on ssDNA using DpnII digestion assay. MA fails to inhibit ALKBH5 demethylation. (**D**) Shown are HPLC traces of FTO demethylation on m^6^A in ssRNA in the absence and presence of the inhibitor MA, respectively. (**E**) Shown are HPLC traces of ALKBH5 demethylation on m^6^A in ssRNA in the absence and presence of the inhibitor MA, respectively.

Both FTO and ALKBH5 use an oxidation reaction to remove the methyl modification of m^6^A in single-stranded nucleic acids (Figure 1A). We set up an FP assay in order to compare the differences in the displacement of dm^6^A-containing ssDNA binding to FTO and ALKBH5, respectively, in the presence of compounds. We defined those compounds as selectively disrupted FTO binding to ssDNA substrate, but these compounds failed to compete on ALKBH5 binding to ssDNA as possible hits. Out of an initial screening of 900 drugs collected randomly at the Shanghai Institute of Materia Medica, three exhibited the selective ability to compete on FTO over ALKBH5 binding to dm^6^A-containing ssDNA at a single dose of concentration (Supplementary Figure S1 and Supplementary Table S1). In the screenings rhein always tested as a positive control. After carefully excluding false positives and non-specific compounds, MA was selected for further validation of its inhibitory activity (Figure 1A). First, we adopted the restriction endonuclease digestion assay to evaluate the inhibitory activity of MA. Reactions were run with 1 μM FTO and 1 μM 49 nt dm^6^A-containing ssDNA and varying concentrations of MA at room temperature for 2 h. Good demethylation activity of the purified FTO enzyme was observed in the control reaction, indicating that FTO was biochemically active (Figure 1B). MA was shown to inhibit FTO demethylation in a dose-dependent manner. The demethylation activity of FTO was completely abolished when 100-fold excess of MA was added to the system with respect to ssDNA substrate (Figure 1B). To confirm the observed inhibitory activity of MA, reaction products were subjected to HPLC-based detection assay. As shown in Supplementary Figure S2, nucleosides dC, dG, dT, dA and dm^6^A were well separated in the HPLC trace, while corresponding dm^6^A absorbent peaks disappeared in the presence of 0.2 equivalent of FTO to ssDNA. This result indicates an FTO-mediated conversion of dm^6^A to normal dA. Treatment with the equivalent of 20 MA to ssDNA under the same reaction conditions effectively abolished FTO demethylation activity, as evidenced by the remaining dm^6^A peak. We have determined the dose-dependent response of MA inhibition of FTO demethylation on a dm^6^A-containing 20 nt ssDNA at pH 7.5 and 25ºC. The IC_50_ value was measured to be 7 μM using the HPLC assay (Supplementary Figure S3A). In addition, the presence of 20 equivalents of inhibitor MA to FTO protein also effectively abolished FTO demethylation on ssRNA, as evidenced by the remaining m^6^A peak in the HPLC trace (Figure 1D). The IC_50_ value for ssRNA inhibition was measured at 8 μM (Supplementary Figure S3B). These *in vitro* biochemical experiments revealed that MA efficiently inhibited FTO demethylation of m^6^A-containing single-stranded nucleic acids.

### Selectivity of the inhibitory activity of MA

Parallel experiments were carried out for comparison. Namely, the inhibitory activity of MA on ALKBH5 demethylation on the same nucleic acid substrate was examined in both restriction endonuclease digestion and HPLC-based detection assay. Both assays first validated that the purified ALKBH5 enzyme was in the active form for dm^6^A demethylation in ssDNA (Figure 1C; Supplementary Figure S4). However, MA could not inhibit the ALKBH5-mediated dm^6^A conversion to adenine in ssDNA even at high concentrations up to 0.5 mM (100-fold compared to ALKBH5) *in vitro* (Figure 1C; Supplementary Figure S4), nor could it inhibit the m^6^A demethylation in ssRNA (Figure 1E). These results show exclusively the selective property of MA inhibition on FTO demethylation of m^6^A substrates over ALKBH5 *in vitro*. In order to further test the *in vitro* target selectivity and the off-target effects of MA on inhibition of nucleic acid demethylation, we tested the inhibition of dm^1^A repair by two other human demethylases, ALKBH2 and ALKBH3 (53), respectively, by using the robust restriction endonuclease digestion assay (Supplementary Figure S5A). As expected, MA could not inhibit ALKBH2 repair dm^1^A in dsDNA (Supplementary Figure S5B); nor does MA inhibit ALKBH3 demethylation of dm^1^A in ssDNA (Supplementary Figure S5C). These results establish MA as a highly selective inhibitor of FTO *in vitro*.

### MA competes on FTO binding to ssDNA

**Figure 2.**
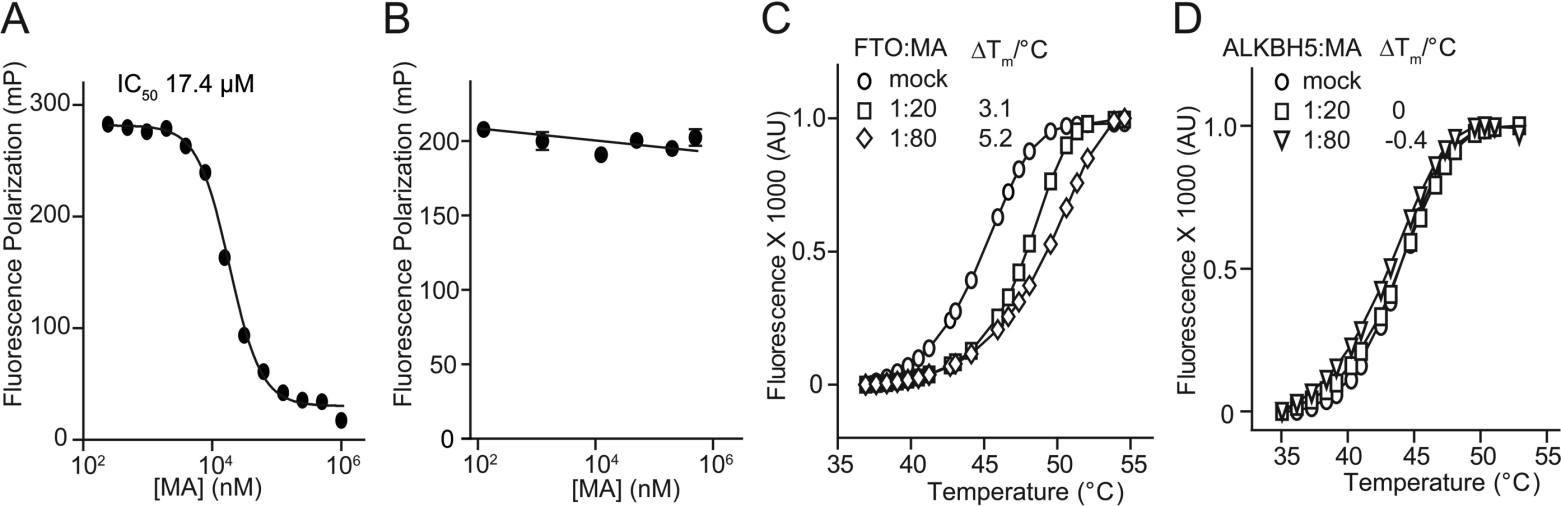
Interaction of MA and m^6^A demethylases. (**A**) Displacement of dm^6^A-containing ssDNA substrates from FTO binding by inhibitor MA. The observed IC_50_ is 17.4 μM for ssDNA competition. (**B**) The ssDNA binding of ALKBH5 stays intact in the presence of MA. (**C**) Thermal shift assay was performed in order to show that MA stabilizes FTO by increasing *T_m_* over 5°C. Unfolding transition of 2 μM FTO in the absence and presence of 20- and 80-folds MA is shown, respectively. (**D**) MA could not shift *T_m_* of the ALKBH5 protein.

We then investigated the interaction between FTO enzyme and inhibitor MA. Following the FP assay, we analyzed competition-binding curves to yield IC_50_ 17.4 μM, thus demonstrating that MA is capable of competing with FTO when binding to ssDNA (Figure 2A). On the contrary, binding between ALKBH5 and ssDNA was slightly disrupted in the presence of 1 mM MA (Figure 2B), which explains the inactivity of MA for the inhibition of ALKBH5 demethylation (Figure 1C and E; Supplementary Figure S4). In order to further elucidate the interaction between FTO and the inhibitor MA, we performed a fluorescence-based thermal shift (DSF) assay to test if MA directly binds to FTO *in vitro*. The purified FTO protein was subjected to a gradual increase in temperature during which the temperature shift between the melting temperature (*T_m_*) in the absence and presence of the bound MA was measured. As Figure 2C shows, the binding of MA significantly stabilized the FTO protein as a reflection of the increasing *T_m_* over 5°C in the presence of 80-fold excess molar of inhibitor MA. This stabilization property of MA on FTO protein was shown in a dose-dependent manner. Although no linear correlation of the degree of temperature shift upon ligand binding with binding affinity could be established (54,55), this high degree of *T_m_* shift of FTO by MA, at least in this one instance, suggests direct binding of the MA inhibitor to the target FTO. Again, in agreement with the observed inactive competition on ALKBH5 binding to ssDNA by MA (Figure 2B), MA minimally alters the thermal stability of ALKBH5 protein, suggesting that MA does not bind to ALKBH5 *in vitro* (Figure 2D). In summary, MA likely competes for FTO binding with dm^6^A-containing ssDNA through a direct interaction with the FTO protein *in vitro*. In contrast, MA neither competes for ALKBH5 binding to ssDNA, nor does it bind directly to the ALKBH5 protein, thus explaining the target selectivity of MA on FTO over ALKBH5 *in vitro*.

### Mechanism of FTO inhibition by MA

**Figure 3.**
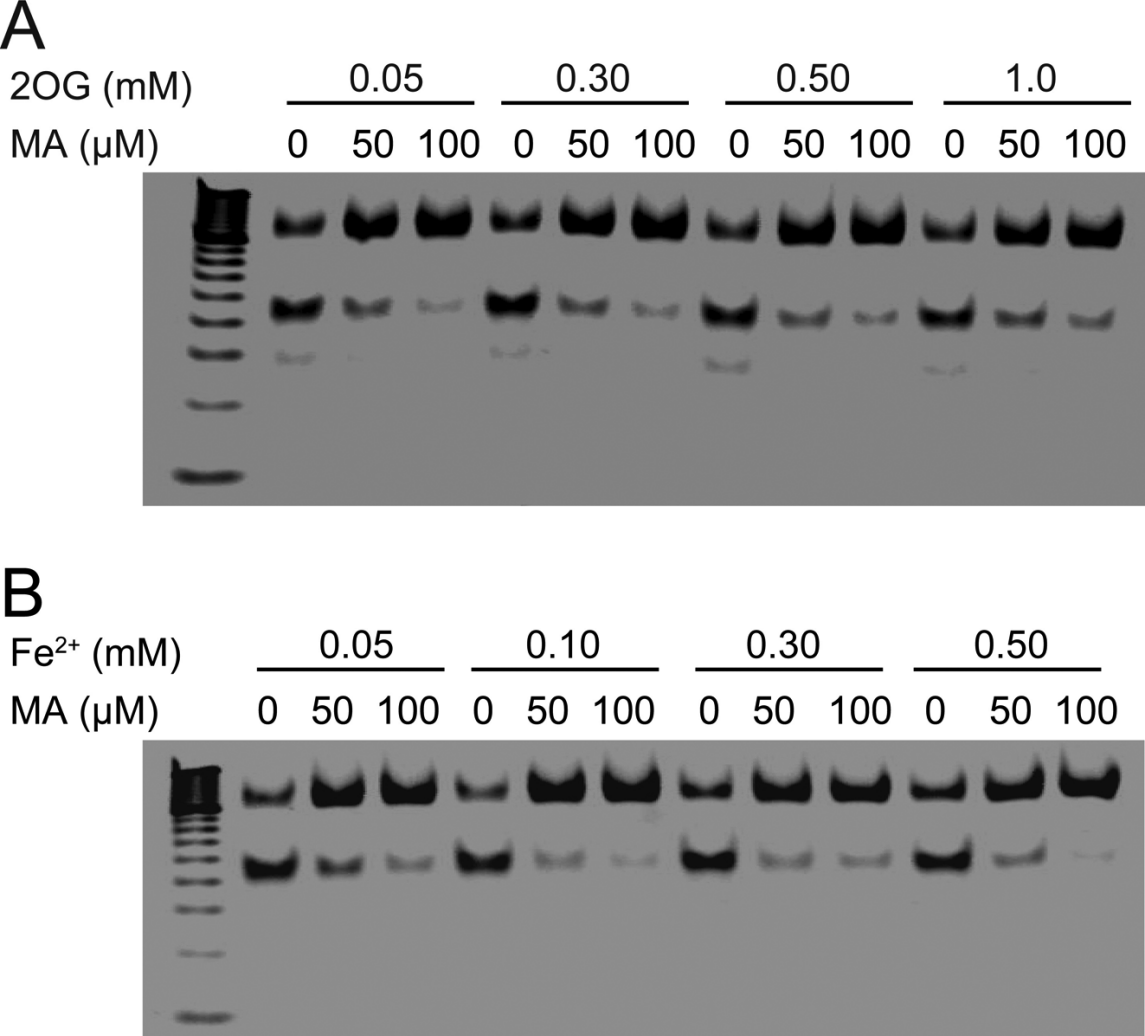
Study of the mechanism of MA inhibition of FTO demethylation. (**A**) The inhibitory activity of MA is independent of the 2OG concentration ranging from 50 μM to 1 mM. (**B**) PAGE assay showing inhibition of FTO demethylation on ssDNA with excess Fe^2+^. All reactions were performed at room temperature in duplicate.

We next focused on a mechanistic study of the inhibition of FTO demethylation of m^6^A in the presence of the inhibitor MA. Earlier structures showed that Arg316 plays an essential role in bridging 2OG binding in the active site of FTO. The carboxyl group in MA might make direct contact with Arg316 through hydrogen-bonding or salt bridge interactions. If so, 2OG could compete with MA for binding in the cofactor-binding site. We carried out inhibitory reactions of FTO demethylation in the presence of MA by varying the 2OG concentrations from 50 μM to 1 mM. As shown in Figure 3A, the inhibitory activity of MA is independent of 2OG at the detected concentrations, suggesting the unlikelihood that MA competes for 2OG binding in the cofactor-binding site. MA is further unlikely to mimic a 2OG derivative in the inhibition mechanism. MA is also known to be capable of chelating divalent metal ions to form coordinated complexes under certain reaction conditions (56). We needed to exclude MA as a Fe^2+^ chelator, in which case it would poison free iron and thus inhibit FTO demethylation. To this end, we performed inhibition reactions by varying the concentrations of free Fe^2+^. Our results reveal that under demethylation conditions, concentrations of Fe^2+^ (Figure 3B) minimally affect the inhibitory activity of MA, thus excluding the possibility that inhibitor MA would chelate free ion. Taken together, MA is neither a chemical mimic of 2OG nor a chelator of iron in the mechanism of inhibition of FTO demethylation *in vitro*.

### Structural insight into the mechanism of the inhibition of FTO by MA

**Figure 4.**
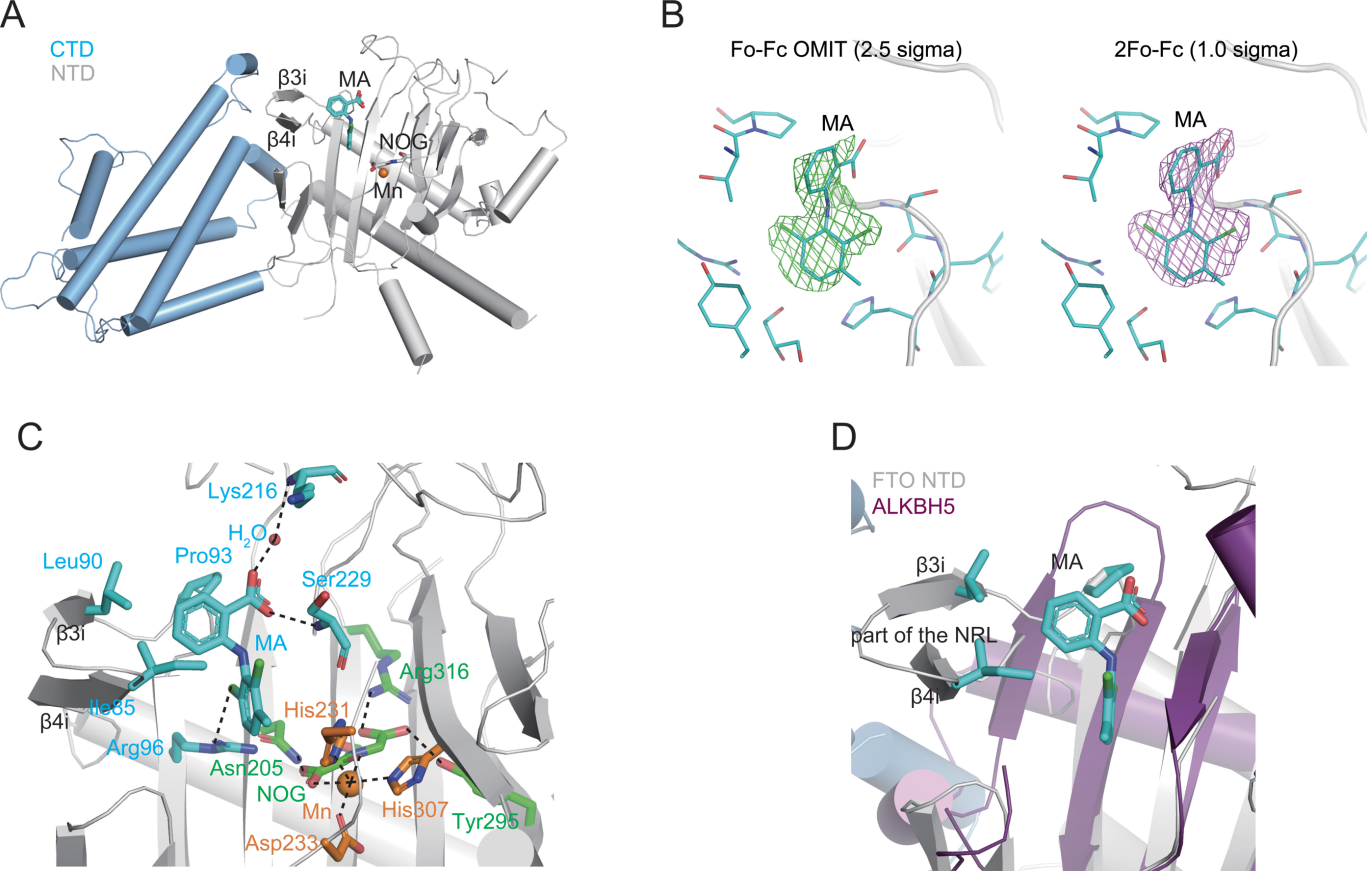
Crystal structure of the FTO/MA complex. (**A**) Overall structure of the complex of MA bound to FTO (PDB code 4QKN) is presented in cartoon. The C-terminal domain (CTD) of FTO is colored in light blue, the N-terminal domain (NTD) in gray, the inhibitor MA in cyan, Mn^2+^ in orange, oxygen atom in red and nitrogen in blue. The part of the NRL consists of two antiparallel β-sheets: β3i and β4i. (**B**) The Fo-Fc OMIT density contoured to 2.5 sigma (left) and 2Fo-Fc density contoured to 1.0 sigma (right) confirm that MA is indeed bound. (**C**) Interaction networks between the FTO protein and MA inhibitor in the presence of cofactor and ion in the active pocket. The environmental residues involved in interactions are shown as sticks. Dark dotted lines indicate the coordination of Mn^2+^ by ligands and hydrogen bondings. (**D**) Superimposition of ALKBH5 (PDB code 4O7X) to CTD of FTO illustrates why MA cannot inhibit ALKBH5 demethylation. ALKBH5 is colored in magenta. The part of the NRL is indicated.

Structural studies of DNA repair enzymes and m^6^A demethylases of AlkB family provide a full profile of substrate recognitions as well as an understanding of cofactor bindings for oxidative demethylation in the highly conserved jelly-roll motif (57–62). However, solely based on these structures (consider FTO and ALKBH5 (46,63–65), for example), the structural elements that account for the selective inhibition of FTO demethylation over ALKBH5 remain unclear. In order to investigate the molecular recognition at atomic resolution, the structure of FTO in complex with the inhibitor MA was determined by crystallography in the presence of metal ion and *N*-Oxalylglycine (NOG), a chemical mimic of cofactor 2OG. The structural complex was solved by molecular replacement using the structure of FTO/dm^3^T complex (PDB code 3LFM) as the searching model, and refined to 2.20 Å resolution (Figure 4A). The final *R*_work_ and *R*_free_ were 19.7 and 23.4%, respectively. Supplementary Table S2 summarizes the data collection as well as refinement statistics. Structural superimposition of the complexes of FTO/MA and FTO/dm^3^T clearly revealed global differences in overall protein folding. As shown in Supplementary Figure S6A, the major differences arise from the two helices, α3 in the N-terminal domain (NTD) and α11 in the C-terminal domain (CTD). This is likely a result of crystal packing and occurs regardless of ligand binding. MA was bound in the NTD region, and close to the interface of NTD and CTD. Electron density maps allow us to clearly assess the presence of the inhibitor. The Fo-Fc OMIT density contoured to 2.5 sigma and 2Fo-Fc density contoured to 1.0 sigma confirm that the inhibitor MA is indeed bound (Figure 4B). The binding site of MA is partially overlaid by dm^3^T- and rhein-binding sites (Supplementary Figure S6B). This explains the competitive property of MA binding to FTO over the m^6^A-containing nucleic acid.

Next, we carefully examined the recognition mode and interaction networks between the inhibitor MA and FTO target in the presence of cofactors (Figure 4C). Typically, cofactors are bound by FTO in a standard conformation resembling most often seen in the complex of FTO/dm^3^T and other AlkB demethylases. The metal ion is ligated by His231, Asp233 and His307, and bidentate NOG exists in an octahedral geometry. The following side chains come into contact with NOG: Asn205, Tyr295 and Arg316 through hydrogen bonding or salt bridge interactions. Of note, FTO recognizes the inhibitor MA extensively. Hydrogen bonding occurs between the carboxyl group in MA and amides in Ser229 directly and Lys216 through a water molecule, respectively. One chlorine atom in MA directly contacts the complex guanidinium group in Arg96. In addition, the phenyl ring bearing carboxyl acid substituent forms hydrophobic interactions with the side chains of neighboring residues, Ile85, Leu90 and Pro93. Through close inspection of other available structures of FTO, we have observed that molecules of related symmetry (residues 33–40 and 339–360) contact the model of FTO in the substrate-binding region where MA binds. As shown in Supplementary Figure S6C, the portion of the molecules with related symmetry in the region of the bound MA demonstrates that MA has no contact with these symmetry-related molecules. Of note, the first loop in the FTO nucleotide recognition lid (NRL), β3i and β4i, which has been defined as the part of the NRL, provides hydrophobic interactions with inhibitor MA. ALKBH5 lacks this part of the NRL loop compared to FTO (46), consequently, thus leading to leakage upon MA binding (Figure 4D). Indeed, this part of the NRL in FTO illustrates the principles behind the selectivity of inhibition of FTO demethylation over ALKBH5. In addition, MA could not inhibit the subfamily members that do have this region of the NRL, including ALKBH2 and ALKBH3 (Supplementary Figure S5). The existence of the hydrophilic and bulky residues in the part of the NRL might significantly disturb the inhibitor MA binding to ALKBH2 or ALKBH3 similarly to FTO (Supplementary Figure S7). Taken together, from the structure of FTO/MA, it should be possible to design analogs that optimize the specificity and selectivity for FTO inhibition.

### Investigation of the structure–activity relationship of inhibitors

**Figure 5.**
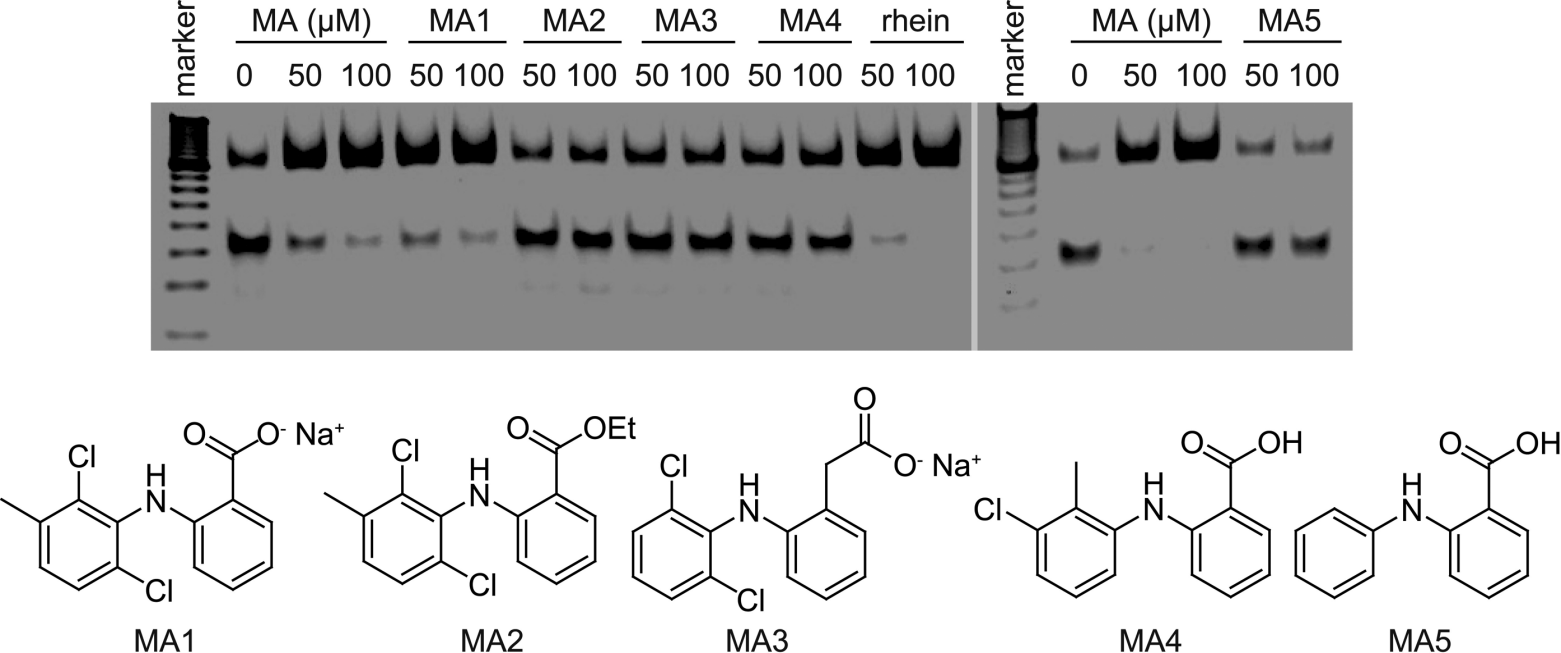
Inhibition of FTO demethylation on ssDNA by MA derivatives detected in PAGE assay. Rhein is tested as a positive control of inhibition. Compounds are examined for their inhibitory activity on 50 and 100 μM, respectively. Chemical structure for each compound is shown.

In order to further understand the recognition mode demonstrated by the structure of FTO/MA, we tested the inhibitory activities of some chemical analogs of the inhibitor MA (Figure 5). All compounds are commercially available, and we do not perform structural optimization at this stage. As expected, the sodium form of inhibitor MA, MA1, still exhibits good inhibitory activity on FTO demethylation *in vitro*. The ester form of MA, compound MA2, completely loses its inhibitory activity *in vitro*, thus revealing the importance of the interactions mediated through the carboxyl group. On the other hand, the ester modification might aid the inhibitor in penetrating cells. Diclofenac sodium, MA3, bearing one-carbon longer carboxyl acid substituent compared to MA, fails to inhibit FTO, also indicating the importance of hydrogen bonding between the carboxyl group and the amides as observed in the structural complex. Why neither MA4 (tolfenamic acid) nor MA5 efficiently inhibits FTO *in vitro* may involve the lack of a chlorine substituent or improper substituted positions, which are essential in maintaining the continued interactions of, or the proper conformation for, compound binding (Supplementary Figure S8). This concise study of the relationships within structure–activity further validates our structural observations, and reveals the competitive and selective mechanism of inhibition on FTO demethylation by the inhibitor MA.

### Inhibitor elevates the levels of cellular m^6^A in mRNA by targeting FTO

**Figure 6.**
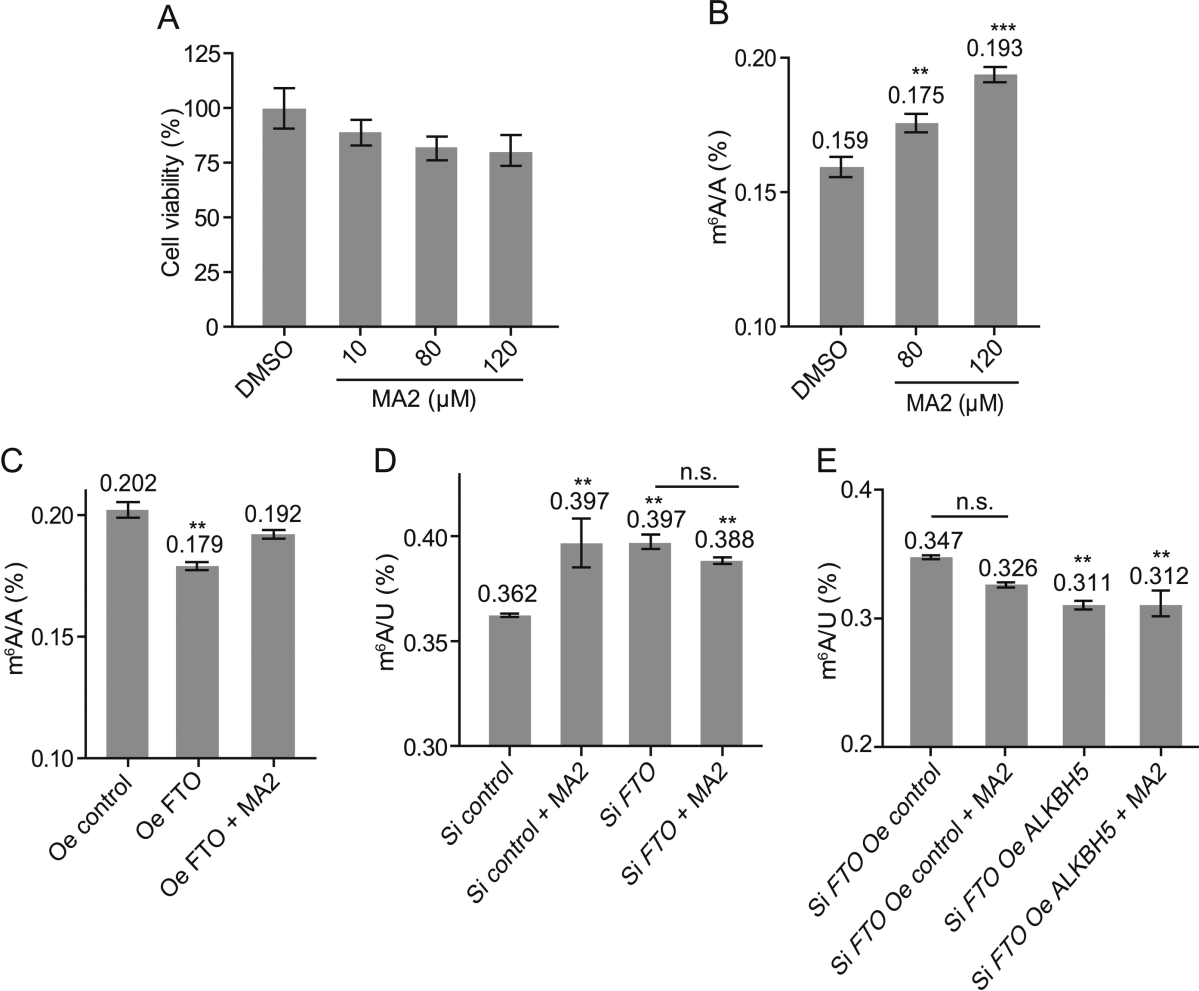
Inhibitor increases the cellular levels of m^6^A modification in HeLa cells. (**A**) MTT assay determines a safe dosage. Values represent mean ± SEM and data are representative of three independent experiments. (**B**) HeLa cells are treated with compound MA2, and the cellular levels of m^6^A are quantified. **P < 0.01, ***P < 0.001, determined using Student's *t*-test. Error bar, mean ± SEM for *n* = 3 experiments. (**C**) Overexpression of FTO and inhibitor regulate m^6^A content in mRNA of HeLa. The m^6^A/A ratio of mRNA samples isolated from HeLa cells with overexpressed (Oe) FTO in the absence and presence of compound MA2, respectively, was quantified by LC-MS/MS. **P < 0.01, determined using Student's *t*-test. Error bar, mean ± SEM for *n* = 3 experiments. (**D**) Knockdown of *FTO* and inhibitor regulates the m^6^A content in mRNA of HeLa. Quantification of the m^6^A/U ratio of mRNA samples isolated from HeLa cells, treated with SiRNA *FTO*, in the presence of compound MA2, was determined by LC-MS/MS, respectively. **P < 0.01, n.s., not significant, determined using Student's *t*-test. Error bar, mean ± SEM for *n* = 3 experiments. (**E**) Overexpression of ALKBH5 and FTO inhibitor regulates m^6^A levels in mRNA in HeLa. Quantification of the m^6^A/U ratio of mRNA samples isolated from SiRNA *FTO* HeLa cells, SiRNA *FTO* HeLa with overexpressed ALKBH5, in the absence or presence of compound MA2, was determined by LC-MS/MS, respectively. **P < 0.01, n.s., not significant, determined using Student's *t*-test. Error bar, mean ± SEM for *n* = 3 experiments.

We then investigated if FTO inhibitor could modulate the level of m^6^A modification in mRNA inside cells. The m^6^A residues in HeLa cells exist in an FTO activity-dependent manner as seen for other cell lines, such as 293FT and BE(2)-C (3,42). Compound MA2, the ethyl ester derivative of MA, is used to achieve better cell penetration. Through the use of LC-MS/MS analysis, our preliminary study revealed that MA2 is hydrolyzed to yield MA in HeLa cells (Supplementary Figure S9). We performed MTT assays to ascertain a safe dosage for cell treatment. >90% of the cells remain viable at 120 μM MA2 as determined by the MTT (Figure 6A). Cells that received treatment of compound MA2 at a concentration of 80 and 120 μM show significant increases in the level of m^6^A modification in total mRNA compared to the untreated control group (Figure 6B).

Next, we investigated the target engagement of FTO inhibitor *in vivo*. We overexpressed FTO using a mammalian expression vector. Western blotting of the total cell lysates from transfected cells confirmed the overexpression of FTO protein after 24 h (Supplementary Figure S10A). As expected, total mRNA isolated from HeLa cells overexpressing the wild-type FTO showed a notable decrease of m^6^A (Figure 6C). Interestingly, cellular m^6^A is restored back to a level comparable to levels seen in the control experiment, when the cells overexpressing the wild-type FTO were treated with 80 μM MA2 (Figure 6C). This effect appears to be at least partially due to the selective *in vivo* inhibition of FTO demethylase. We also transfected HeLa cells with *FTO* siRNA, and western blotting of the total cell lysates from transfected cells showed the knockdown of FTO 24 h post-transfection (Supplementary Figure S10B). Consistent with previous observations, our result shows that knockdown of the cellular FTO increases m^6^A in mRNA (Figure 6D). Of note, the treatment of the transfected HeLa cells with *FTO* siRNA with compound MA2 does not further alter the cellular level of m^6^A, indicating that MA2 lacks the ability to inhibit the demethylations of m^6^A through ALKBH5 or any unidentified demethylases (Figure 6D).

Lastly, we performed further *in vivo* studies of the selective inhibitory effect of FTO on ALKBH5. We overexpressed ALKBH5 in transfected HeLa cells with *FTO* siRNA using a mammalian expression vector. Western blotting of the total cell lysates from transfected cells confirmed the overexpression of ALKBH5 and the knockdown of FTO protein after 24 h (Supplementary Figure S10B). As shown in Figure 6E, overexpression of ALKBH5 in the *FTO* knockdown cells decreases the cellular level of m^6^A in mRNA with significant differences, indicating that ALKBH5 is the active form of demethylation. The treatment of MA2 does not noticeably affect the total level of m^6^A in HeLa cells of *FTO* knockdown when ALKBH5 demethylase is overexpressed (Figure 6E). We concluded that the increase of cellular m^6^A is a result of the selective inhibition of FTO over ALKBH5 demethylation due to the presence of the small-molecule inhibitor *in vivo*.

## DISCUSSION

Methylation of nucleic acids has two forms: damage and modification. 5-methylcytosine is the epigenetic marker of DNA. mRNA modification represents another layer of epigenetics, analogous to DNA methylation and histone modification in the regulation of gene expression (29). Increasing evidence shows that m^6^A in mRNA may affect mRNA splicing, trafficking, translation and stability, thus establishing that the reversible m^6^A modification in mRNA plays broad and critical roles in fundamental biological processes and disease development (66). To date, two AlkB family demethylases, FTO and ALKBH5, have been identified to efficiently convert m^6^A to adenosine (3,27). ‘RNA epigenetics’, centered on m^6^A modification, has become a fast-moving research field in chemical biology and holds promise for future therapeutic development. Future studies will focus on its fundamental roles and its dynamic regulation of m^6^A in specific biological contexts. Unlike siRNA that removes an entire protein, the selective inhibitor only shuts down the activity of certain targets. Small molecule-inhibiting FTO is of importance, therefore, and may prove to have a profound impact on the study of the fundamental biology of mRNA demethylation as well as related enzymes.

We have identified rhein as the first cell-active inhibitor of m^6^A demethylation by applying a structure-based virtual screening (42). This result has paved the way for the development of functional probes to target m^6^A demethylases for mRNA on the cellular level. Rhein displays moderate inhibitory selectivity for nucleic acid demethylation in the AlkB subfamily, however. The structure of FTO/rhein has revealed the inhibitor mode of action *in vitro* (44). Recently, novel dihydroxyfuran sulfonamides have been identified as FTO inhibitors through design rationale (in order to mimic ascorbic acid), which show anticonvulsant activity (41). In addition, the 2-OG tethering strategy has also been successfully applied to the development of selective inhibitor of FTO *in vitro* (45). These studies provide both competitive and non-competitive inhibitors *in vitro*, which will aid the development of more potent and specific FTO inhibitors. However, reported compounds have not yet been demonstrated to selectively inhibit FTO over ALKBH5 *in vivo*.

We screened a library of older drugs using a high-throughput FP assay for compounds that compete with FTO to bind to dm^6^A-containing ssDNA. We performed experimental validation to identify MA as a new class of inhibitor, which is selective for FTO demethylation over ALKBH5 *in vitro* (Figure 1). MA inhibits FTO demethylation of an m^6^A-containing ssDNA or ssRNA in a dose-dependent manner; results of the HPLC assay measured the IC_50_ value at 7 and 8 μM, respectively (Supplementary Figure S3). However, MA fails to inhibit the ALKBH5-mediated conversion of m^6^A to adenine either in ssDNA or in ssRNA even at high concentrations (IC_50_ > 0.5 mM) (Figure 1C and E; Supplementary Figure S4). MA could not inhibit dm^1^A repair in dsDNA by ALKBH2, or in ssDNA by ALKBH3 *in vitro* (Supplementary Figures S5 and S7). These results therefore establish MA as a highly selective inhibitor of FTO demethylation *in vitro*. MA competes for FTO binding with m^6^A-containing ssDNA likely through direct interaction with the FTO protein. In contrast, MA could not bind to the ALKBH5 protein; nor does it compete for ALKBH5 binding to ssDNA, thus explaining the target selectivity of MA on FTO over ALKBH5 (Figure 2). Further biochemical experiments show that MA is neither a chemical mimic of 2OG nor a chelator of iron in the mechanism of inhibition of FTO demethylation (Figure 3). Of note, the structural complex of MA bound to FTO reveals a β-hairpin motif that is a part of the NRL for providing extra interactions between MA and FTO (Figure 4). ALKBH5 lacks this region of the part of the NRL however, which results in leakage when binding to MA. From this structural complex, it should be possible to design analogs that optimize specificity and potency for targeting FTO exclusively. Finally, we also investigate if MA could modulate cellular levels of m^6^A residues in mRNA in an FTO-activity-dependent manner (Figure 6). Further study of the *in vivo* selective inhibition of FTO over ALKBH5 is also performed, revealing that MA could be a functional probe of FTO demethylation in RNA epigenetics.

MA is a non-steroidal anti-inflammatory drug; it is primarily known for its inhibition of prostaglandins synthesis as well as for its more specific inhibition of cyclooxygenase enzymes and lipoxygenases (67), which may be of interest because recently identified FTO inhibitors are also derived from dual cyclooxygenase/lipoxygenase inhibitors (41). While any possible connections between anti-inflammatory and selective inhibition of FTO or m^6^A modification remain to be explored, the identification of MA as a highly selective inhibitor of FTO will stimulate more research into specific inhibitors that exist within our resources of known drugs, for example other cyclooxygenase inhibitors and members of the class of ascorbic acid-derived inhibitors. Such studies might even link the regulation of m^6^A modification on gene expression to human diseases.

In summary, we report here on MA, an inhibitor of FTO demethylation of m^6^A over ALKBH5. MA is shown to efficiently and selectively inhibit FTO demethylation by competition on m^6^A-containing substrate binding. The structural complex of MA bound to FTO provides molecular insight into the mechanism of competitive inhibition of FTO, explains why MA selectively inhibits FTO over ALKBH5 and also reveals a novel binding site to address the challenge of selectivity in the future design of specific inhibitors of FTO. In addition, the regulation of m^6^A abundance through the inhibitor MA in HeLa cells occurs in an FTO activity-dependent manner, thus revealing the target engagement of cellular FTO over ALKBH5. Our work sheds light on the development of new chemical probes for studies on the roles of human m^6^A demethylases in epigenetic processes and presents possibilities for therapeutic leads for target validation in drug discovery.

## ACCESSION NUMBERS

Atomic coordinates and structure factors have been deposited in the Protein Data Bank (PDB; www.pdb.org) under accession ID code 4QKN for the structure of FTO/MA.

## SUPPLEMENTARY DATA

Supplementary Data are available at NAR Online.

SUPPLEMENTARY DATA
