# Supplementary material for: Meclofenamic acid selectively inhibits FTO demethylation of m6A over ALKBH5
Source: Nucleic Acids Res. 2014 Dec 1;43(1):373–84. doi: 10.1093/nar/gku1276 (PMC4288171; doi:10.1093/nar/gku1276)
Supplement: SUPPLEMENTARY DATA [file supp_43_1_373__index.html]

Meclofenamic acid selectively inhibits FTO demethylation of m6A over ALKBH5 — Meclofenamic acid selectively inhibits FTO demethylation of m6A over ALKBH5 — SUPPLEMENTARY DATA 

# Meclofenamic acid selectively inhibits FTO demethylation of m6A over ALKBH5

## SUPPLEMENTARY DATA

**Files in this Data Supplement:**

- SUPPLEMENTARY DATA
