# Supplementary material for: Meclofenamic acid selectively inhibits FTO demethylation of m6A over ALKBH5
Source: Nucleic Acids Res. 2014 Dec 1;43(1):373–84. doi: 10.1093/nar/gku1276 (PMC4288171; doi:10.1093/nar/gku1276)
Supplement: SUPPLEMENTARY DATA [file supp_gku1276_nar-01613-a-2014-File008.pdf]

Supplementary data for

Meclofenamic acid selectively inhibits FTO demethylation of m^6A over ALKBH5

Yue Huang[1,†], Jingli Yan[2,†], Qi Li[1], Jiafei Li[1], Shouzhe Gong[1], Hu Zhou[1], Jianhua Gan[3], Hualiang Jiang[4], Gui-Fang Jia[2,*], Cheng Luo[4,*], and Cai-Guang Yang[1,*]

[1] Chinese Academy of Sciences Key Laboratory of Receptor Research, Shanghai Institute of Materia Medica, Chinese Academy of Sciences, Shanghai 201203, China

[2] Synthetic and Functional Biomolecules Center, Beijing National Laboratory for Molecular Sciences, Key Laboratory of Bioorganic Chemistry and Molecular Engineering of Ministry of Education, College of Chemistry and Molecular Engineering, Peking University, Beijing 100871, China

[3] School of Life Sciences, Fudan University, Shanghai 200433, China

[4] State Key Laboratory of Drug Research, Shanghai Institute of Materia Medica, Chinese Academy of Sciences, Shanghai 201203, China

*To whom correspondence should be addressed. Tel: +86 21 50806029; Fax: +86 21 50807088; Email: yangcg@simm.ac.cn
Correspondence should also be addressed to Cheng Luo. Tel: +86 21 50271399; Fax: +86 21 50807188; Email: cluo@simm.ac.cn
Correspondence should also be addressed to Guifang Jia. Tel: +86 10 62756179; Fax: +86 10 62754637; Email: guifangjia@pku.edu.cn

[†]The authors wish it to be known that, in their opinion, the first two authors should be regarded as Joint First Authors.

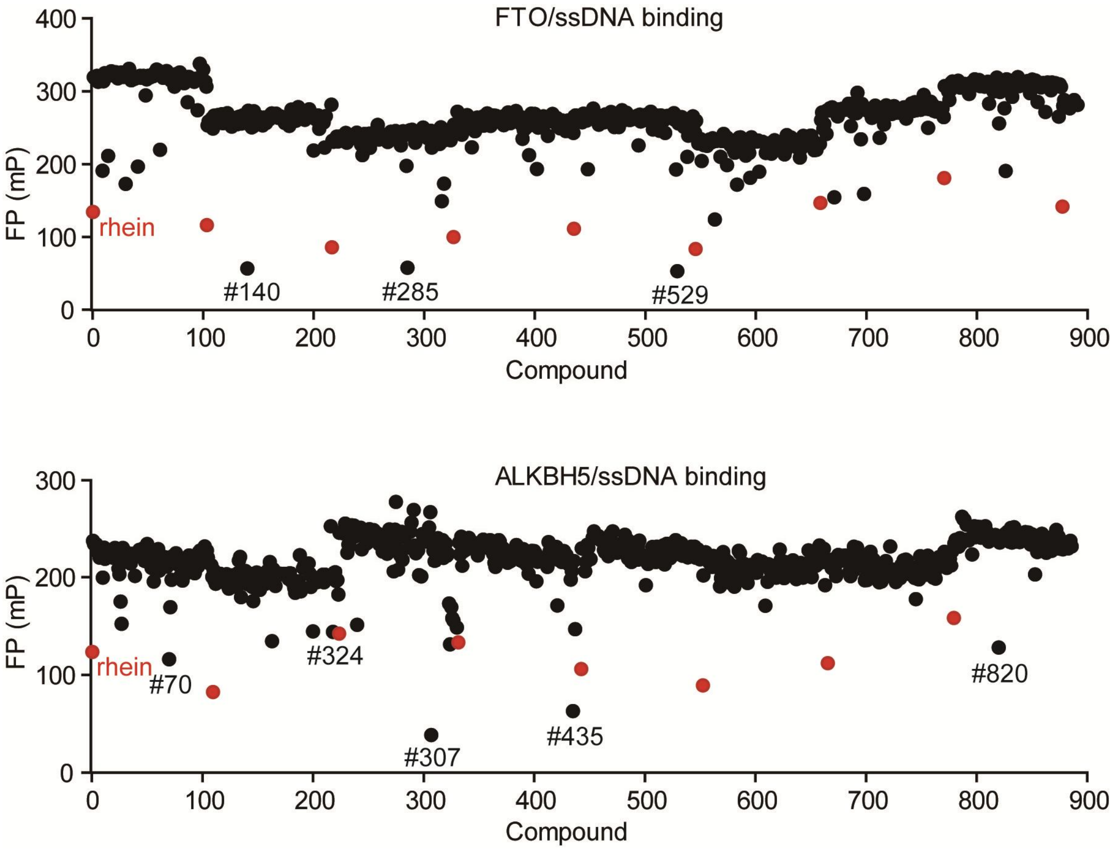

Figure S1. High-throughput screening of inhibitors selectively disrupt FTO/ssDNA interaction by using an FP assay. Rhein is tested for positive control, and labeled with a red dot. Compounds that show comparable or better inhibitory activities than rhein are labeled accordingly with an ID.

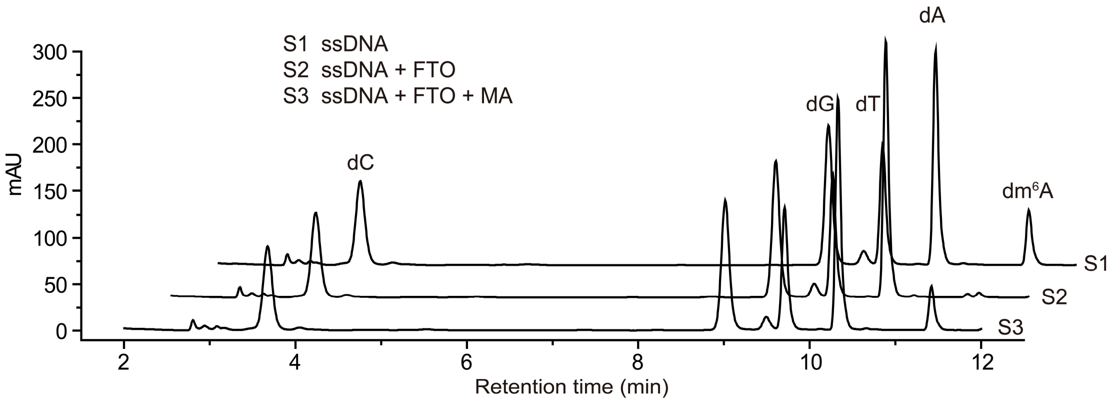

Figure S2. Shown are HPLC traces of FTO demethylation on dm^{6}A in ssDNA in the absence and presence of the inhibitor MA, respectively.

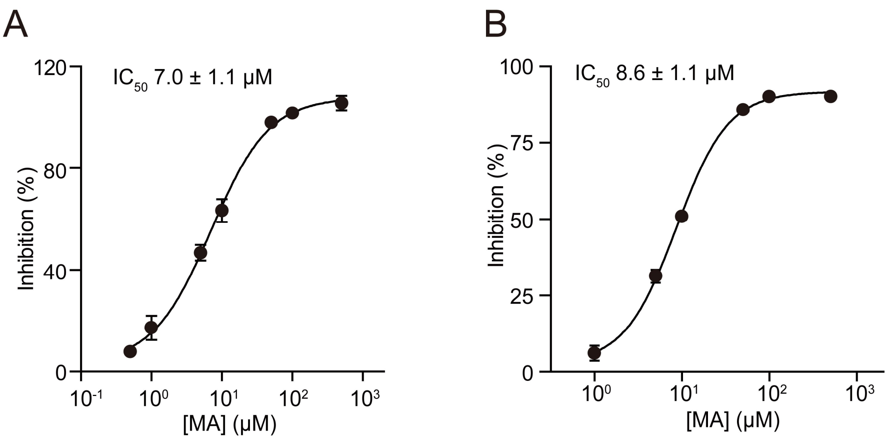

Figure S3. (A) Quantification of the inhibition of MA on FTO demethylation of dm^{6}A in ssDNA recorded in HPLC traces. Each reaction is assayed in triplicate, and the standard deviation is indicated. (B) Quantification of inhibitory activity of MA on FTO demethylation of m^{6}A in ssRNA recorded in HPLC traces. Each reaction is assayed in triplicate, and the standard deviation is indicated.

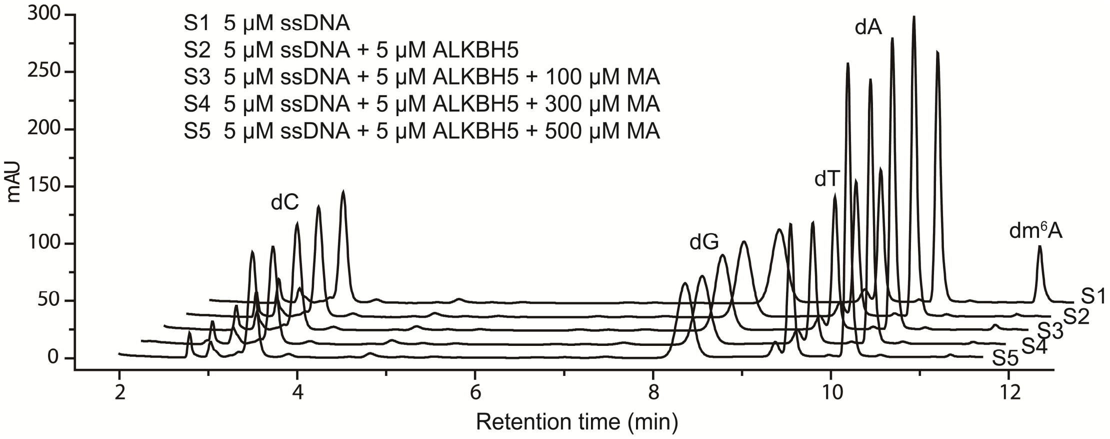

Figure S4. Shown are HPLC traces of ALKBH5 demethylation of dm^{6}A-containing ssDNA in the absence and presence of the inhibitor MA at varying concentrations, respectively. The assignments of dC, dG, dT, dA, and dm^{6}A are indicated in the HPLC trace.

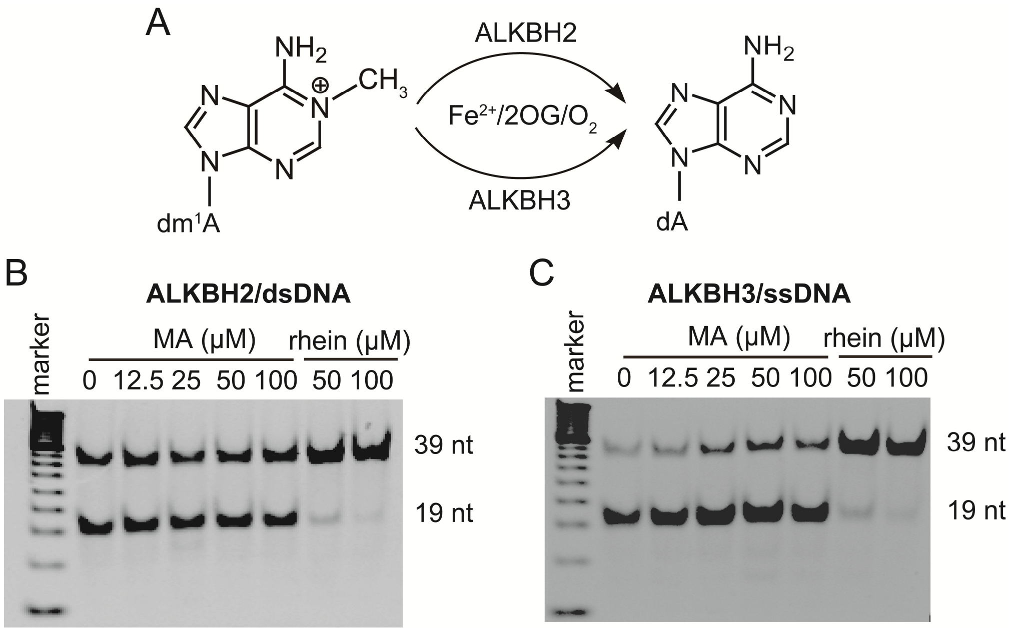

Figure S5. MA can not inhibit dm^{1}A repair by ALKBH2 or ALKBH3 *in vitro*. (A) Scheme of dm^{1}A repair by ALKBH2 and ALKBH3. (B) Detection of inhibition of ALKBH2-processed dm^{1}A repair in dsDNA using the restriction enzyme digestion assay. In PAGE image, the upper band is 39 nt dsDNA with dm^{1}A incorporation, and the lower band represents the demethylated products after DpnII digestion. In the control experiment, rhein inhibits ALKBH2 in a dose-response manner. MA is inhibitory inactive on ALKBH2 repair of dm^{1}A in dsDNA. (C) Detection of MA inhibition of ALKBH3 demethylation of dm^{1}A in ssDNA using DpnII digestion assay. In the control experiment, rhein inhibits ALKBH3 in a dose-response manner.

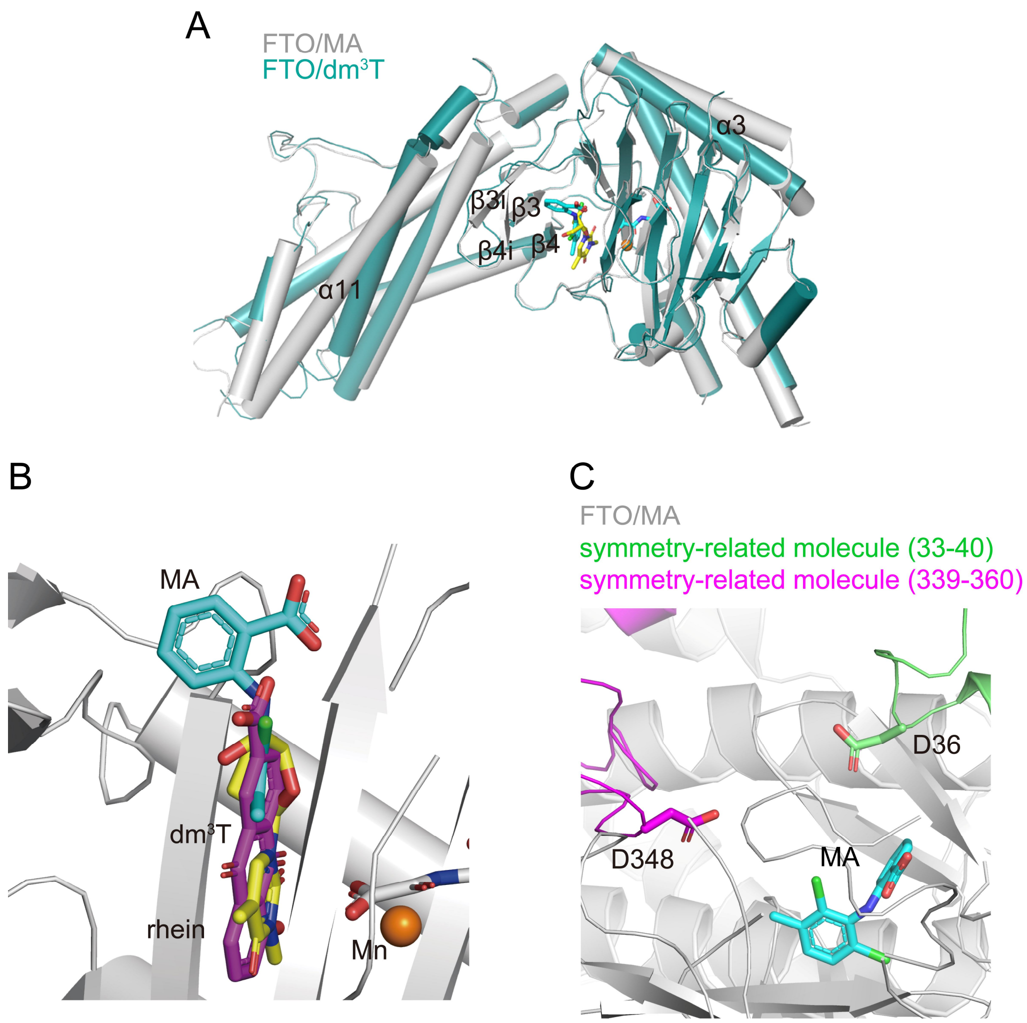

Figure S6. MA bound to FTO. (A) Cartoon presentation of the superimposition of two complexes performed in PyMOL. FTO/MA (PDB code 4QKN) is colored in gray, FTO/dm^{3}T (PDB code 3LFM) in light blue, MA in cyan, and dm^{3}T in yellow, respectively. The structure variants α3 and α11 are indicated. (B) Structural alignment of FTO complexes with different ligands bound to the nucleoside recognition pocket. Structural alignment was performed in PyMOL with the structures of FTO/MA (PDB code 4QKN), FTO/rhein (PDB code 4IE7), and FTO/dm^{3}T (PDB code 3LFM). Ligands are shown as sticks, and rhein is colored in magenta, dm^{3}T in yellow. (C) The portion of the symmetry-related molecules (residues 33-40 and 339-360) in the region of the bound MA shows no contacts that MA may have with these symmetry-related molecules.

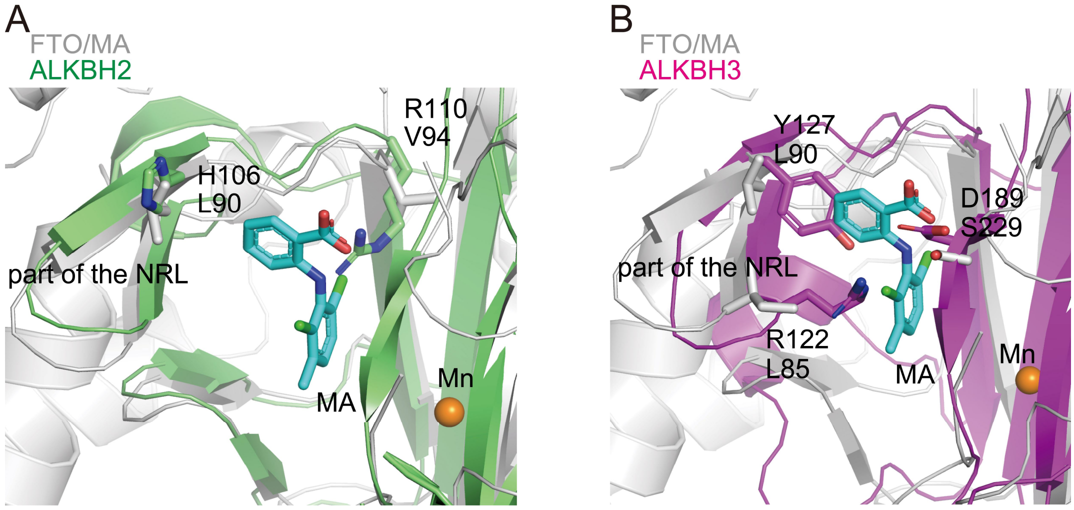

Figure S7. Structural features that determine the selectivity of inhibitor MA in AlkB subfamily. (A) The superimposition of FTO/MA (PDB code 4QKN) and ALKBH2 (PDB code 3BUC) performed in PyMOL. FTO/MA was colored in gray, ALKBH2 in green, MA in cyan, and Mn in orange, respectively. Residues H106 and R110 are from ALKBH2, and L90 and V94 are from FTO/MA. (B) The superimposition of FTO/MA and ALKBH3 (PDB code 2IUW) performed in PyMOL. FTO/MA was colored in gray, ALKBH3 in magenta, MA in cyan, and Mn in orange, respectively. Residues R122, Y127, and D189 are from ALKBH3, and L85, L90, and S229 are from FTO/MA.

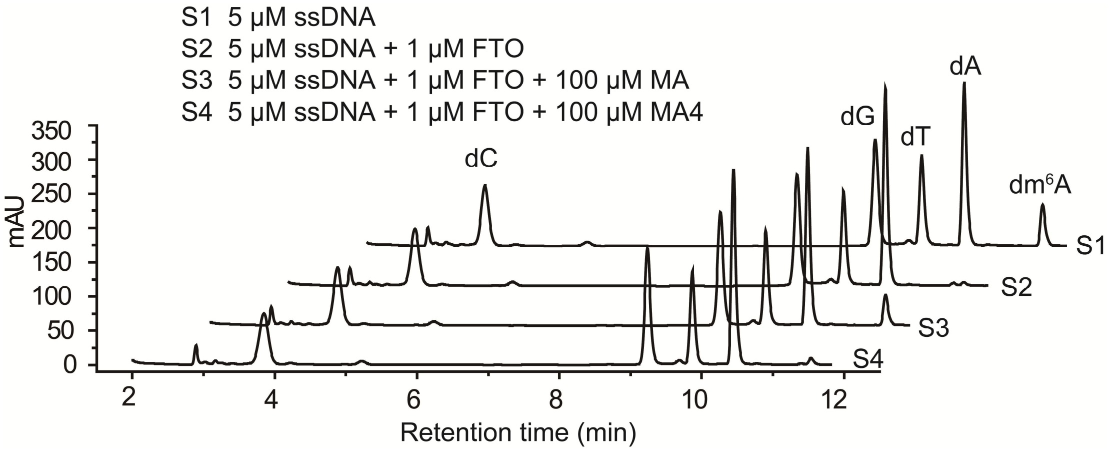

Figure S8. Shown are HPLC traces of FTO demethylation of dm^{6}A-containing ssDNA in the presence of inhibitor MA and MA4, respectively. The assignments of dC, dG, dT, dA, and dm^{6}A are indicated in the HPLC trace.

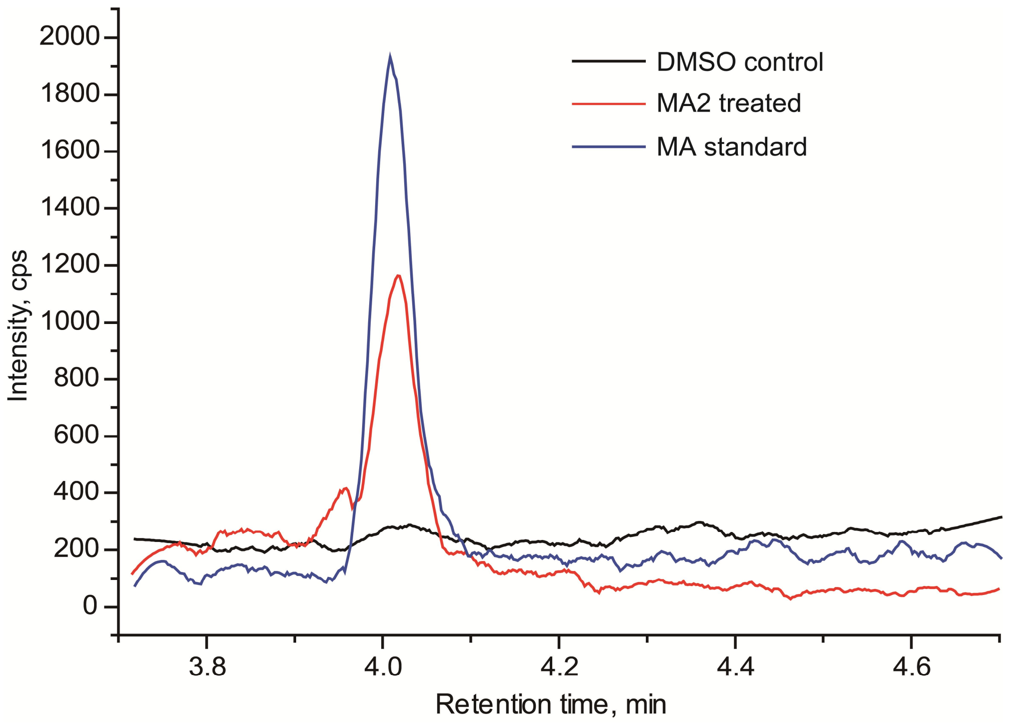

Figure S9. LC-MS/MS determination of the hydrolysis of MA2 to yield MA in HeLa cells. HeLa cells are treated with 80 μM of MA2 and incubated at 37 °C for 24 h. The cells are then lysed and the extract analyzed for the presence and the hydrolysis product of MA2, MA, using LC-MS/MS analysis. The compounds are identified by MS/MS characterization and by comparing their retention time with that of standard.

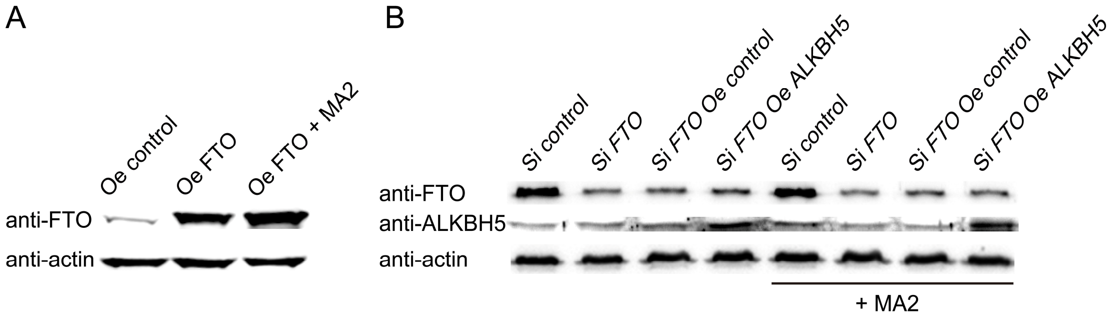

Figure S10. (A) Western blot quantification of FTO protein in HeLa cells with overexpressed FTO. (B) Western blot quantification of FTO and ALKBH5 protein in HeLa cells with SiRNA FTO and overexpressed ALKBH5 with or without FTO inhibitor.

Table S1 List of fluorescence polarization for each compound for both FTO and ALKBH5.

FTO		ALKBH5	
**Compound ID**	**Fluorescence polarization (mP)**	**Compound ID**	**Fluorescence polarization (mP)**
rhein	134.6	rhein	123.5
LCOD2001	319.3	LCOD2001	237.9
LCOD2002	317.6	LCOD2002	234.7
LCOD2004	321.1	LCOD2004	234.4
LCOD2005	313	LCOD2005	231.4
LCOD2007	317.4	LCOD2007	220.6
LCOD2008	318.9	LCOD2008	221.8
LCOD2009	191.1	LCOD2009	200.5
LCOD2010	313.6	LCOD2010	199.6
LCOD2011	324.8	LCOD2011	230.8
LCOD2012	319.6	LCOD2012	219.3
LCOD2014	211.2	LCOD2014	213.4
LCOD2016	326.1	LCOD2016	225.6
LCOD2017	327.3	LCOD2017	227.3
LCOD2018	321.5	LCOD2018	221.7
LCOD2019	323.4	LCOD2019	220.5
LCOD2020	326	LCOD2020	226.3
LCOD2021	326.1	LCOD2021	230.7
LCOD2022	321.2	LCOD2022	221.5
LCOD2023	317.7	LCOD2023	220
LCOD2024	319.2	LCOD2024	209
LCOD2025	319.8	LCOD2025	203.1
LCOD2026	325.7	LCOD2026	175.1
LCOD2027	319.7	LCOD2027	152.3
LCOD2028	319.7	LCOD2028	230.5
LCOD2029	319.6	LCOD2029	226
LCOD2030	172.8	LCOD2030	225.3
LCOD2031	325.6	LCOD2031	226.1
LCOD2032	320	LCOD2032	223.4
LCOD2033	330.7	LCOD2033	219.3
LCOD2035	315.1	LCOD2035	214
LCOD2036	321.2	LCOD2036	224.7
LCOD2037	319	LCOD2037	222.5
LCOD2038	317.1	LCOD2038	220.9
LCOD2039	317.6	LCOD2039	201.4
LCOD2041	196.7	LCOD2041	205.6
LCOD2043	321	LCOD2043	228.5
LCOD2044	318.3	LCOD2044	226

LCOD2045	319.5	LCOD2045	227.4
LCOD2046	321.1	LCOD2046	229.8
LCOD2048	294.3	LCOD2048	216.6
LCOD2049	316.4	LCOD2049	227.6
LCOD2050	320.9	LCOD2050	234.6
LCOD2051	319.6	LCOD2051	218.3
LCOD2052	318.8	LCOD2052	222.7
LCOD2054	320.3	LCOD2054	209.7
LCOD2055	320.7	LCOD2055	215.5
LCOD2056	325.1	LCOD2056	195.7
LCOD2057	324.4	LCOD2057	224.8
LCOD2058	329.6	LCOD2058	206.1
LCOD2059	328.7	LCOD2059	222
LCOD2060	322.8	LCOD2060	229.3
LCOD2061	219.7	LCOD2061	206.9
LCOD2062	326.2	LCOD2062	212.9
LCOD2064	318.4	LCOD2064	220.9
LCOD2066	325.5	LCOD2066	221.2
LCOD2067	327.6	LCOD2067	213.5
LCOD2068	320.7	LCOD2068	210.8
LCOD2069	319.4	LCOD2069	217.7
LCOD2070	317.2	LCOD2070	116.2
LCOD2071	314.5	LCOD2071	169.3
LCOD2072	317.8	LCOD2072	197.4
LCOD2073	316.8	LCOD2073	222.2
LCOD2074	306.1	LCOD2074	216.6
LCOD2075	312.6	LCOD2075	223.1
LCOD2076	323.6	LCOD2076	225
LCOD2077	319.1	LCOD2077	220.4
LCOD2078	322.6	LCOD2078	212.8
LCOD2079	325.7	LCOD2079	218.8
LCOD2080	323.1	LCOD2080	201
LCOD2081	317.6	LCOD2081	216.1
LCOD2082	312.6	LCOD2082	196.7
LCOD2083	311.1	LCOD2083	215.8
LCOD2085	314.4	LCOD2085	203.9
LCOD2086	284.8	LCOD2086	213.7
LCOD2087	319.8	LCOD2087	211.5
LCOD2088	317.5	LCOD2088	222.8
LCOD2090	317	LCOD2090	214.8
LCOD2092	313	LCOD2092	212.9
LCOD2093	317	LCOD2093	204.1
LCOD2094	315.7	LCOD2094	220.6

LCOD2095	273.7	LCOD2095	213.4
LCOD2096	316.7	LCOD2096	210.9
LCOD2097	337.6	LCOD2097	226.7
LCOD2098	318.4	LCOD2098	227.8
LCOD2099	317.8	LCOD2099	215.9
LCOD2100	329.7	LCOD2100	222.6
LCOD2102	313.1	LCOD2102	232
LCOD2103	306.4	LCOD2103	216.6
rhein	116.4	LCOD2104	227.9
LCOD2104	253.3	LCOD2105	220.7
LCOD2105	258.4	LCOD2106	214.3
LCOD2106	258.8	LCOD2108	212.9
LCOD2108	262.7	LCOD2109	200.3
LCOD2109	248.8	rhein	82.6
LCOD2110	266.5	LCOD2110	207.9
LCOD2111	265	LCOD2111	196.6
LCOD2112	266.9	LCOD2112	193.3
LCOD2113	261.6	LCOD2113	202.3
LCOD2114	267.5	LCOD2114	200.8
LCOD2115	262.1	LCOD2115	204.1
LCOD2116	261.2	LCOD2116	196.3
LCOD2117	268.5	LCOD2117	199.8
LCOD2118	256.3	LCOD2118	200.5
LCOD2120	259.1	LCOD2120	199.6
LCOD2121	260.7	LCOD2121	195.8
LCOD2122	269.9	LCOD2122	203
LCOD2123	257.3	LCOD2123	191.2
LCOD2124	265.6	LCOD2124	188.3
LCOD2125	262.4	LCOD2125	189.9
LCOD2126	251.4	LCOD2126	197.4
LCOD2127	254	LCOD2127	201.9
LCOD2128	263.6	LCOD2128	199.6
LCOD2131	254.7	LCOD2131	190.1
LCOD2132	264.9	LCOD2132	204
LCOD2133	265	LCOD2133	217.6
LCOD2134	256.9	LCOD2134	221.4
LCOD2135	255.9	LCOD2135	179.6
LCOD2136	266.8	LCOD2136	197.4
LCOD2138	260.4	LCOD2138	201.9
LCOD2139	264.6	LCOD2139	206.9
LCOD2140 (MA)	57	LCOD2140 (MA)	200.4
LCOD2141	273.3	LCOD2141	192.9
LCOD2142	266.9	LCOD2142	203.1

LCOD2144	250.6	LCOD2144	198.3
LCOD2145	251.6	LCOD2145	190
LCOD2146	272.6	LCOD2146	175.6
LCOD2147	262.7	LCOD2147	203
LCOD2148	265.3	LCOD2148	196.9
LCOD2149	264.7	LCOD2149	205.4
LCOD2150	265	LCOD2150	198
LCOD2151	262.7	LCOD2151	197
LCOD2152	256.8	LCOD2152	187.1
LCOD2153	258.4	LCOD2153	194.8
LCOD2154	253	LCOD2154	192.3
LCOD2155	263.7	LCOD2155	200.3
LCOD2156	260.1	LCOD2156	200.4
LCOD2157	256.6	LCOD2157	193.4
LCOD2158	262	LCOD2158	198.8
LCOD2159	264.8	LCOD2159	207.1
LCOD2160	261.5	LCOD2160	202.6
LCOD2161	267.6	LCOD2161	216.1
LCOD2162	269.6	LCOD2162	206.4
LCOD2164	267.2	LCOD2164	208.6
LCOD2165	264.9	LCOD2165	199.4
LCOD2166	269.3	LCOD2166	191.2
LCOD2168	263.4	LCOD2168	204.1
LCOD2170	265.5	LCOD2170	204.2
LCOD2172	264	LCOD2172	203
LCOD2174	256.6	LCOD2174	203.9
LCOD2178	252.5	LCOD2178	198.3
LCOD2179	266.2	LCOD2179	204.6
LCOD2180	274.8	LCOD2180	197.6
LCOD2181	266.8	LCOD2181	195.4
LCOD2183	270.5	LCOD2183	189.1
LCOD2184	259.6	LCOD2184	184.2
LCOD2185	266.2	LCOD2185	191.6
LCOD2186	278.1	LCOD2186	195.9
LCOD2187	267.1	LCOD2187	195.9
LCOD2188	265.7	LCOD2188	223.2
LCOD2189	268.5	LCOD2189	185.6
LCOD2191	267.1	LCOD2191	209.5
LCOD2192	260.7	LCOD2192	212.6
LCOD2196	275.2	LCOD2196	214.7
LCOD2197	271.2	LCOD2197	203.9
LCOD2198	266.6	LCOD2198	190.1
LCOD2199	266.7	LCOD2199	200.9

LCOD2200	218.8	LCOD2200	144.6
LCOD2201	263.2	LCOD2201	199.5
LCOD2202	261.6	LCOD2202	198.3
LCOD2203	265.1	LCOD2203	197.7
LCOD2204	266.9	LCOD2204	195.2
LCOD2205	248.3	LCOD2205	197.5
LCOD2207	257.7	LCOD2207	200.4
LCOD2209	257.2	LCOD2209	202.7
LCOD2210	222.7	LCOD2210	194.8
LCOD2211	265.7	LCOD2211	202.7
LCOD2216	281.4	LCOD2216	252.9
rhein	86.1	LCOD2217	200.9
LCOD2217	232.1	LCOD2218	144
LCOD2218	230.8	LCOD2220	205.7
LCOD2220	235.5	LCOD2221	196
LCOD2221	237.7	LCOD2222	197
LCOD2222	231.1	LCOD2223	182.4
LCOD2223	236.6	rhein	142.3
LCOD2224	238.2	LCOD2224	246
LCOD2228	232.4	LCOD2228	245.3
LCOD2229	242.7	LCOD2229	255.5
LCOD2230	229.4	LCOD2230	253.1
LCOD2231	235.1	LCOD2231	225.6
LCOD2232	233.3	LCOD2232	234.1
LCOD2233	239.6	LCOD2233	232.9
LCOD2235	237	LCOD2235	253.9
LCOD2236	239.2	LCOD2236	243.2
LCOD2237	236.3	LCOD2237	253.7
LCOD2238	235.6	LCOD2238	242.3
LCOD2239	243.2	LCOD2239	245.8
LCOD2240	238.5	LCOD2240	151.4
LCOD2241	236.8	LCOD2241	243.9
LCOD2243	226.9	LCOD2243	251.4
LCOD2244	212.3	LCOD2244	229
LCOD2246	241.3	LCOD2246	247.8
LCOD2247	240	LCOD2247	242
LCOD2248	240.6	LCOD2248	234.9
LCOD2249	230.7	LCOD2249	235.6
LCOD2250	241.8	LCOD2250	248.6
LCOD2251	222.5	LCOD2251	249.9
LCOD2255	241.7	LCOD2255	248.9
LCOD2256	237.7	LCOD2256	246.3
LCOD2257	247.4	LCOD2257	239.3

LCOD2258	253.9	LCOD2258	245.5
LCOD2259	233.8	LCOD2259	244.8
LCOD2260	242.6	LCOD2260	234.4
LCOD2262	239	LCOD2262	233.8
LCOD2263	234.4	LCOD2263	226.8
LCOD2264	230.5	LCOD2264	228.3
LCOD2267	236.4	LCOD2267	224.6
LCOD2268	232.6	LCOD2268	241.7
LCOD2269	244.6	LCOD2269	249.5
LCOD2270	236.8	LCOD2270	240.3
LCOD2271	239.2	LCOD2271	244.6
LCOD2272	234.7	LCOD2272	235.9
LCOD2273	245.4	LCOD2273	206
LCOD2274	239.9	LCOD2274	249.3
LCOD2275	236.8	LCOD2275	277.9
LCOD2276	240.2	LCOD2276	230.3
LCOD2277	237	LCOD2277	207.9
LCOD2278	225.8	LCOD2278	227.4
LCOD2279	238.2	LCOD2279	222.8
LCOD2280	245.1	LCOD2280	247.2
LCOD2281	240	LCOD2281	218.4
LCOD2284	197.7	LCOD2284	245.9
LCOD2285	58	LCOD2285	95.2
LCOD2287	246.3	LCOD2287	230.8
LCOD2288	244.7	LCOD2288	247.3
LCOD2289	247.1	LCOD2289	256.7
LCOD2291	239.8	LCOD2291	269.5
LCOD2292	229.3	LCOD2292	233
LCOD2293	242	LCOD2293	225.1
LCOD2295	241.1	LCOD2295	228
LCOD2296	244.1	LCOD2296	202.2
LCOD2298	242.4	LCOD2298	200.9
LCOD2299	250.1	LCOD2299	244.9
LCOD2300	238.2	LCOD2300	240.5
LCOD2302	240.2	LCOD2302	238
LCOD2303	235.8	LCOD2303	237
LCOD2304	238.3	LCOD2304	247.3
LCOD2305	239.8	LCOD2305	251.5
LCOD2306	245.8	LCOD2306	267.3
LCOD2307	222.8	LCOD2307	38.7
LCOD2308	237	LCOD2308	234.6
LCOD2309	241.4	LCOD2309	227.7
LCOD2310	243.1	LCOD2310	216.9

LCOD2311	238	LCOD2311	232.7
LCOD2312	240.4	LCOD2312	236.2
LCOD2313	246.8	LCOD2313	240.6
LCOD2314	227.8	LCOD2314	235.3
LCOD2315	250.4	LCOD2315	223.7
LCOD2316	149.2	LCOD2316	210.2
LCOD2317	242.3	LCOD2317	238.6
LCOD2318	173.4	LCOD2318	220.3
LCOD2319	233.2	LCOD2319	235.9
LCOD2320	242.7	LCOD2320	235.1
LCOD2321	237.4	LCOD2321	222.5
LCOD2322	241.5	LCOD2322	224.7
LCOD2323	244	LCOD2323	173.1
LCOD2324	232.6	LCOD2324	131.4
LCOD2325	246.3	LCOD2325	169.4
LCOD2326	243.6	LCOD2326	158.2
rhein	100	LCOD2327	155.7
LCOD2327	254.3	LCOD2330	148.7
LCOD2330	271.7	rhein	133.4
LCOD2332	258.5	LCOD2332	236
LCOD2333	263.4	LCOD2333	223.9
LCOD2334	240.6	LCOD2334	242
LCOD2335	248	LCOD2335	211.8
LCOD2336	257.9	LCOD2336	227
LCOD2337	257.5	LCOD2337	228.7
LCOD2338	254.6	LCOD2338	228.6
LCOD2339	267.1	LCOD2339	232.3
LCOD2340	263.8	LCOD2340	236.7
LCOD2341	261.8	LCOD2341	240.8
LCOD2342	260.8	LCOD2342	235.2
LCOD2343	222.9	LCOD2343	232.1
LCOD2344	261.7	LCOD2344	236.7
LCOD2346	245.2	LCOD2346	233.8
LCOD2348	263.8	LCOD2348	231.5
LCOD2349	256.9	LCOD2349	222.9
LCOD2350	254.5	LCOD2350	230.9
LCOD2351	261.6	LCOD2351	228.8
LCOD2352	260.5	LCOD2352	230.9
LCOD2353	265.1	LCOD2353	231.8
LCOD2354	252.8	LCOD2354	239.1
LCOD2355	258	LCOD2355	238.2
LCOD2356	265.3	LCOD2356	238.1
LCOD2357	269.4	LCOD2357	236.9

LCOD2358	259.9	LCOD2358	231.4
LCOD2360	252.4	LCOD2360	238.5
LCOD2361	257.1	LCOD2361	228.7
LCOD2362	266.8	LCOD2362	227.8
LCOD2363	262.3	LCOD2363	224.4
LCOD2364	266.6	LCOD2364	215.4
LCOD2365	266	LCOD2365	210.5
LCOD2366	259	LCOD2366	219.6
LCOD2367	262.3	LCOD2367	237.5
LCOD2368	261.8	LCOD2368	238.5
LCOD2369	261.1	LCOD2369	237.2
LCOD2371	265	LCOD2371	226.7
LCOD2372	253.5	LCOD2372	230.2
LCOD2373	266.4	LCOD2373	219.4
LCOD2374	262.5	LCOD2374	222.3
LCOD2375	262.1	LCOD2375	216.1
LCOD2376	270.7	LCOD2376	214.9
LCOD2378	256.2	LCOD2378	216.1
LCOD2379	264.7	LCOD2379	223.1
LCOD2380	265.3	LCOD2380	224.3
LCOD2381	261.3	LCOD2381	227.4
LCOD2383	272.7	LCOD2383	228.5
LCOD2384	263.5	LCOD2384	233.7
LCOD2387	255.9	LCOD2387	225.1
LCOD2388	264.1	LCOD2388	228.7
LCOD2389	234.9	LCOD2389	216.5
LCOD2390	246.9	LCOD2390	229.3
LCOD2392	249.8	LCOD2392	220.8
LCOD2393	259.6	LCOD2393	213
LCOD2394	258.2	LCOD2394	214
LCOD2395	212.4	LCOD2395	203.7
LCOD2397	269.2	LCOD2397	217.9
LCOD2398	253.9	LCOD2398	227.7
LCOD2400	269	LCOD2400	227.4
LCOD2401	266.1	LCOD2401	225.4
LCOD2402	193.3	LCOD2402	195.9
LCOD2403	250	LCOD2403	215.8
LCOD2404	257.5	LCOD2404	216.4
LCOD2405	253.6	LCOD2405	220.5
LCOD2406	260.2	LCOD2406	219.6
LCOD2408	265.6	LCOD2408	219.4
LCOD2409	263.5	LCOD2409	219.6
LCOD2410	256.4	LCOD2410	215.4

LCOD2412	238.7	LCOD2412	210.6
LCOD2413	264.5	LCOD2413	236.7
LCOD2414	261	LCOD2414	227.7
LCOD2415	261.5	LCOD2415	228.9
LCOD2416	268.8	LCOD2416	224.4
LCOD2419	256	LCOD2419	219
LCOD2420	263.1	LCOD2420	231.7
LCOD2421	252.2	LCOD2421	171
LCOD2422	251.9	LCOD2422	221.8
LCOD2423	249	LCOD2423	220
LCOD2424	252.7	LCOD2424	223.9
LCOD2425	259.7	LCOD2425	215.6
LCOD2426	259.1	LCOD2426	226.5
LCOD2427	257.7	LCOD2427	227.4
LCOD2428	245.4	LCOD2428	221.7
LCOD2429	253.9	LCOD2429	223.4
LCOD2430	259.9	LCOD2430	217.5
LCOD2431	253.9	LCOD2431	213.3
LCOD2432	245.2	LCOD2432	216.9
LCOD2433	259.1	LCOD2433	197.5
LCOD2434	255	LCOD2434	206.4
LCOD2435	242.7	LCOD2435	63
rhein	111.53	LCOD2436	217
LCOD2436	265.4	LCOD2437	146.9
LCOD2437	267	rhein	106.1
LCOD2443	257.5	LCOD2443	230
LCOD2444	269.2	LCOD2444	229.7
LCOD2446	261.7	LCOD2446	206.2
LCOD2447	259.7	LCOD2447	215.9
LCOD2448	193.1	LCOD2448	233.8
LCOD2449	269.2	LCOD2449	230.5
LCOD2451	265.5	LCOD2451	219.2
LCOD2453	276.3	LCOD2453	241.2
LCOD2454	265.2	LCOD2454	247.7
LCOD2456	264.9	LCOD2456	242.9
LCOD2457	264.4	LCOD2457	240.3
LCOD2458	254	LCOD2458	242.9
LCOD2460	266.2	LCOD2460	238.7
LCOD2462	260.4	LCOD2462	237
LCOD2463	257.5	LCOD2463	228.5
LCOD2464	268.3	LCOD2464	226.3
LCOD2465	261.1	LCOD2465	235.9
LCOD2466	261	LCOD2466	233.2

LCOD2467	264.1	LCOD2467	222
LCOD2468	261.2	LCOD2468	234
LCOD2469	271.4	LCOD2469	244.4
LCOD2470	265.7	LCOD2470	238.2
LCOD2471	261.6	LCOD2471	247.6
LCOD2472	250.9	LCOD2472	235.1
LCOD2473	267.1	LCOD2473	233.2
LCOD2474	268.2	LCOD2474	233.7
LCOD2475	260.4	LCOD2475	225.5
LCOD2476	250.8	LCOD2476	228
LCOD2477	257.7	LCOD2477	229.2
LCOD2478	264.4	LCOD2478	229.5
LCOD2479	274	LCOD2479	227.1
LCOD2480	262.9	LCOD2480	228.5
LCOD2481	266.5	LCOD2481	236.9
LCOD2482	260.8	LCOD2482	244.2
LCOD2483	270.4	LCOD2483	235.4
LCOD2484	266	LCOD2484	239.3
LCOD2485	263.1	LCOD2485	219.9
LCOD2486	260.5	LCOD2486	221.8
LCOD2487	262.1	LCOD2487	222.4
LCOD2488	261.5	LCOD2488	215.6
LCOD2491	264.7	LCOD2491	236.2
LCOD2492	264	LCOD2492	233.6
LCOD2493	266.6	LCOD2493	218
LCOD2494	225.8	LCOD2494	232.4
LCOD2495	267.8	LCOD2495	238.2
LCOD2496	258.5	LCOD2496	224.7
LCOD2497	266.9	LCOD2497	221.8
LCOD2499	260.3	LCOD2499	222.2
LCOD2500	265.5	LCOD2500	221
LCOD2501	268	LCOD2501	191.8
LCOD2502	262.2	LCOD2502	215.9
LCOD2503	264.2	LCOD2503	213.6
LCOD2507	247.6	LCOD2507	231.7
LCOD2509	264.4	LCOD2509	222.6
LCOD2510	266.7	LCOD2510	215.6
LCOD2511	263.9	LCOD2511	231.9
LCOD2512	255.4	LCOD2512	229.5
LCOD2513	245.7	LCOD2513	227.6
LCOD2514	266.8	LCOD2514	223.4
LCOD2515	261.9	LCOD2515	222.6
LCOD2516	259.8	LCOD2516	221.6

LCOD2517	260.6	LCOD2517	233.3
LCOD2518	242.7	LCOD2518	213.1
LCOD2519	263.3	LCOD2519	228.2
LCOD2520	266	LCOD2520	234.8
LCOD2522	265.3	LCOD2522	227.2
LCOD2523	265.6	LCOD2523	220.1
LCOD2525	267.2	LCOD2525	231.6
LCOD2526	270	LCOD2526	229.2
LCOD2527	260.6	LCOD2527	223.8
LCOD2528	192.5	LCOD2528	238.7
LCOD2529	53.2	LCOD2529	218.4
LCOD2530	255.7	LCOD2530	231.8
LCOD2532	264.5	LCOD2532	233.2
LCOD2533	264.4	LCOD2533	235.3
LCOD2534	261.5	LCOD2534	222.7
LCOD2535	251.2	LCOD2535	222.5
LCOD2536	256.8	LCOD2536	225.7
LCOD2538	210.1	LCOD2538	225.7
LCOD2539	239.2	LCOD2539	220.9
LCOD2540	257.2	LCOD2540	229.5
LCOD2541	254.8	LCOD2541	224.9
LCOD2542	259.3	LCOD2542	231.9
LCOD2543	265.6	LCOD2543	225.8
LCOD2544	255.6	LCOD2544	230.1
LCOD2545	242.8	LCOD2545	231.9
LCOD2546	259.6	LCOD2546	220.6
rhein	83.6	LCOD2547	227
LCOD2547	236.3	LCOD2548	229.1
LCOD2548	228.5	LCOD2549	227.7
LCOD2549	230.6	LCOD2550	218.8
LCOD2550	234.4	LCOD2551	226.4
LCOD2551	204.3	rhein	89.3
LCOD2553	226	LCOD2553	201.8
LCOD2554	232.2	LCOD2554	223.8
LCOD2555	231.4	LCOD2555	226
LCOD2556	225.2	LCOD2556	224.2
LCOD2557	230.7	LCOD2557	218.8
LCOD559	233.9	LCOD2559	214.2
LCOD2560	230	LCOD2560	217.4
LCOD2561	235.4	LCOD2561	210.6
LCOD2562	233.4	LCOD2562	222.3
LCOD2563	124.2	LCOD2563	210.7
LCOD2564	231.6	LCOD2564	215.9

LCOD2566	232.8	LCOD2566	217.2
LCOD2567	232.8	LCOD2567	209.4
LCOD2568	210	LCOD2568	190.5
LCOD2569	231.9	LCOD2569	199
LCOD2570	231.8	LCOD2570	195.6
LCOD2571	241.3	LCOD2571	226.4
LCOD2572	234.5	LCOD2572	219.9
LCOD2574	198.8	LCOD2574	208.3
LCOD2575	230.3	LCOD2575	216.4
LCOD2576	233.6	LCOD2576	214.8
LCOD2577	226.2	LCOD2577	203.6
LCOD2578	231.6	LCOD2578	193.9
LCOD2579	229.6	LCOD2579	212.4
LCOD2580	219.3	LCOD2580	205.5
LCOD2581	215.8	LCOD2581	190.4
LCOD2582	227.6	LCOD2582	196
LCOD2583	171.9	LCOD2583	206.7
LCOD2584	227.9	LCOD2584	208.2
LCOD2585	236.4	LCOD2585	227.7
LCOD2586	236.7	LCOD2586	225.3
LCOD2588	228.1	LCOD2588	214.8
LCOD2590	227	LCOD2590	214
LCOD2591	211.8	LCOD2591	205.6
LCOD2592	229.2	LCOD2592	198
LCOD2593	227.2	LCOD2593	194.1
LCOD2594	215.5	LCOD2594	209.9
LCOD2595	181.2	LCOD2595	202.9
LCOD2596	236.8	LCOD2596	213
LCOD2597	230.9	LCOD2597	208
LCOD2599	230.6	LCOD2599	211
LCOD2601	231.1	LCOD2601	204.6
LCOD2602	232.5	LCOD2602	218.1
LCOD2603	189.5	LCOD2603	210.6
LCOD2606	230.6	LCOD2606	216.5
LCOD2609	215.4	LCOD2609	170.9
LCOD2611	228.4	LCOD2611	195.3
LCOD2612	221	LCOD2612	213.8
LCOD2613	219	LCOD2613	209.6
LCOD2614	214.4	LCOD2614	205.7
LCOD2615	227.6	LCOD2615	212.3
LCOD2616	231.3	LCOD2616	205.6
LCOD2617	232.8	LCOD2617	201.9
LCOD2618	230.4	LCOD2618	211.5

LCOD2619	222.5	LCOD2619	199.7
LCOD2621	226.3	LCOD2621	213.8
LCOD2622	228.7	LCOD2622	228.8
LCOD2623	226.9	LCOD2623	218.2
LCOD2624	228.3	LCOD2624	214.5
LCOD2625	220.1	LCOD2625	209.2
LCOD2626	213.4	LCOD2626	199.2
LCOD2627	218.4	LCOD2627	216
LCOD2628	239.9	LCOD2628	211.2
LCOD2629	233.1	LCOD2629	209
LCOD2631	231.9	LCOD2631	207
LCOD2632	236.4	LCOD2632	203
LCOD2633	233.3	LCOD2633	207.8
LCOD2634	232.1	LCOD2634	217.9
LCOD2635	226.4	LCOD2635	204.8
LCOD2636	227.5	LCOD2636	211.1
LCOD2637	227.4	LCOD2637	198.2
LCOD2638	222.5	LCOD2638	208.2
LCOD2639	228.6	LCOD2639	200.1
LCOD2640	208.9	LCOD2640	202.8
LCOD2641	221.3	LCOD2641	215.1
LCOD2642	234.3	LCOD2642	208.9
LCOD2643	238.9	LCOD2643	199.5
LCOD2645	236.4	LCOD2645	212.1
LCOD2647	227.3	LCOD2647	209
LCOD2648	235.5	LCOD2648	228.1
LCOD2649	238.9	LCOD2649	208.8
LCOD2650	239.2	LCOD2650	217.5
LCOD2651	234	LCOD2651	200
LCOD2653	218.9	LCOD2653	221.8
LCOD2658	227.8	LCOD2658	233.2
rhein	147	LCOD2659	215.2
LCOD2659	260.6	LCOD2660	225.9
LCOD2660	270.9	LCOD2661	221.2
LCOD2661	248.3	LCOD2662	229.7
LCOD2662	254.9	LCOD2663	191.9
LCOD2663	241.5	LCOD2664	215.3
LCOD2664	273.2	rhein	112
LCOD2666	273.6	LCOD2666	215.3
LCOD2667	276.6	LCOD2667	213.5
LCOD2669	278	LCOD2669	209.4
LCOD2670	274.3	LCOD2670	218.6
LCOD2671	154.5	LCOD2671	211.4

LCOD2672	273.5	LCOD2672	206.5
LCOD2673	269.3	LCOD2673	201.1
LCOD2674	265.5	LCOD2674	221.9
LCOD2675	275.3	LCOD2675	223.3
LCOD2676	272.8	LCOD2676	216.4
LCOD2678	276	LCOD2678	222.6
LCOD2680	275.3	LCOD2680	217
LCOD2681	274.4	LCOD2681	222.9
LCOD2683	276.5	LCOD2683	221
LCOD2684	282.9	LCOD2684	214.9
LCOD2685	276.8	LCOD2685	205.1
LCOD2686	252	LCOD2686	201.2
LCOD2687	273.3	LCOD2687	206.1
LCOD2688	270.7	LCOD2688	205.8
LCOD2689	283.8	LCOD2689	231.8
LCOD2691	269.8	LCOD2691	216.8
LCOD2692	297.9	LCOD2692	230.8
LCOD2693	278.1	LCOD2693	218.5
LCOD2694	279.2	LCOD2694	221.1
LCOD2695	234.2	LCOD2695	223.8
LCOD2696	282.4	LCOD2696	218
LCOD2697	277.8	LCOD2697	211.4
LCOD2698	159.4	LCOD2698	205.6
LCOD2699	275.3	LCOD2699	196.9
LCOD2700	273.8	LCOD2700	200.7
LCOD2701	275.4	LCOD2701	197
LCOD2702	272.1	LCOD2702	201
LCOD2703	274.9	LCOD2703	220.2
LCOD2704	274	LCOD2704	226.7
LCOD2705	263.6	LCOD2705	221.1
LCOD2706	277.4	LCOD2706	216.1
LCOD2707	275.4	LCOD2707	218.3
LCOD2708	280.1	LCOD2708	214.5
LCOD2709	277.1	LCOD2709	207.8
LCOD2711	274.7	LCOD2711	204.9
LCOD2712	236	LCOD2712	194.9
LCOD2713	275.6	LCOD2713	196.4
LCOD2714	269.4	LCOD2714	195.7
LCOD2715	268.9	LCOD2715	198.3
LCOD2716	254.4	LCOD2716	214.1
LCOD2717	275.8	LCOD2717	212.8
LCOD2718	280.7	LCOD2718	217.2
LCOD2719	272.9	LCOD2719	211.3

LCOD2722	271.5	LCOD2722	232.1
LCOD2723	269	LCOD2723	201.6
LCOD2724	276.7	LCOD2724	208.4
LCOD2725	279.5	LCOD2725	206.2
LCOD2727	270.5	LCOD2727	195.1
LCOD2729	273.7	LCOD2729	203.1
LCOD2730	275.1	LCOD2730	206.2
LCOD2731	270.3	LCOD2731	199.1
LCOD2732	275.8	LCOD2732	213.1
LCOD2734	277.9	LCOD2734	217.9
LCOD2736	262.4	LCOD2736	218.6
LCOD2737	276.6	LCOD2737	217.9
LCOD2738	277.6	LCOD2738	203.6
LCOD2741	283.1	LCOD2741	209.4
LCOD2742	272.7	LCOD2742	203.8
LCOD2744	285	LCOD2744	199.4
LCOD2745	273.8	LCOD2745	177.5
LCOD2746	280.2	LCOD2746	210.9
LCOD2747	276	LCOD2747	198
LCOD2748	277.9	LCOD2748	197.8
LCOD2749	274.6	LCOD2749	222.2
LCOD2750	280.8	LCOD2750	213.3
LCOD2751	282.1	LCOD2751	215.6
LCOD2752	294.9	LCOD2752	210.7
LCOD2754	276.2	LCOD2754	202.5
LCOD2756	249.7	LCOD2756	222.5
LCOD2757	275.5	LCOD2757	202.7
LCOD2758	285.4	LCOD2758	208.8
LCOD2760	281.3	LCOD2760	211.4
LCOD2761	273.1	LCOD2761	199.7
LCOD2762	277.3	LCOD2762	203.8
LCOD2763	272.3	LCOD2763	198.6
LCOD2766	276.9	LCOD2766	221.1
LCOD2767	273.9	LCOD2767	212.7
LCOD2768	271.3	LCOD2768	217.7
LCOD2769	276.2	LCOD2769	219
LCOD2770	264.4	LCOD2770	218.5
rhein	181	LCOD2771	226.6
LCOD2771	306.9	LCOD2773	209.5
LCOD2773	306.9	LCOD2774	225.2
LCOD2774	300	LCOD2775	217.1
LCOD2775	287.9	LCOD2776	219.3
LCOD2776	301.7	LCOD2777	212

LCOD2777	311.9	LCOD2778	227
LCOD2778	313.4	rhein	158.3
LCOD2780	311.6	LCOD2780	235.1
LCOD2781	310.8	LCOD2781	238.9
LCOD2782	309	LCOD2782	235.7
LCOD2783	314.3	LCOD2783	236.7
LCOD2784	307	LCOD2784	231.9
LCOD2785	304.9	LCOD2785	240.5
LCOD2786	308	LCOD2786	235
LCOD2787	310.1	LCOD2787	262.7
LCOD2788	308.6	LCOD2788	260.1
LCOD2789	310.1	LCOD2789	259.7
LCOD2790	308.6	LCOD2790	239
LCOD2791	308.6	LCOD2791	254.4
LCOD2793	296.2	LCOD2793	238.3
LCOD2796	311.3	LCOD2796	223.8
LCOD2797	309.9	LCOD2797	243
LCOD2798	316.4	LCOD2798	252.9
LCOD2799	308.3	LCOD2799	241.7
LCOD2800	311.8	LCOD2800	241.9
LCOD2801	313.1	LCOD2801	252.4
LCOD2802	315.3	LCOD2802	251.5
LCOD2803	306.9	LCOD2803	252
LCOD2804	312.1	LCOD2804	241.2
LCOD2807	306.4	LCOD2807	247.5
LCOD2808	310.1	LCOD2808	252.8
LCOD2809	312.3	LCOD2809	243.8
LCOD2810	312.9	LCOD2810	246.4
LCOD2811	282.9	LCOD2811	237.1
LCOD2815	299.6	LCOD2815	240.3
LCOD2817	311.9	LCOD2817	243.8
LCOD2818	318.7	LCOD2818	242.1
LCOD2820	255.9	LCOD2820	128
LCOD2821	298.9	LCOD2821	236.1
LCOD2822	307.3	LCOD2822	237.3
LCOD2824	308.8	LCOD2824	241.2
LCOD2825	276.6	LCOD2825	241.4
LCOD2826	190.7	LCOD2826	245
LCOD2827	316.2	LCOD2827	245.4
LCOD2828	307.7	LCOD2828	247.1
LCOD2829	311.8	LCOD2829	239.7
LCOD2830	311.7	LCOD2830	236.9
LCOD2831	314.5	LCOD2831	251.2

LCOD2832	292.1	LCOD2832	236.1
LCOD2833	308.4	LCOD2833	244.5
LCOD2834	309.9	LCOD2834	251.9
LCOD2835	309.6	LCOD2835	244.1
LCOD2836	309.3	LCOD2836	237
LCOD2837	319.4	LCOD2837	246.2
LCOD2838	306.9	LCOD2838	245.2
LCOD2839	307.6	LCOD2839	236.6
LCOD2840	309.2	LCOD2840	237.4
LCOD2841	311.9	LCOD2841	239.4
LCOD2843	309.1	LCOD2843	245.2
LCOD2844	310.2	LCOD2844	246.9
LCOD2845	311.7	LCOD2845	242.4
LCOD2846	313.8	LCOD2846	241
LCOD2847	303.6	LCOD2847	246.4
LCOD2848	297.3	LCOD2848	244.1
LCOD2849	310.5	LCOD2849	234.2
LCOD2850	316	LCOD2850	244.6
LCOD2851	307.3	LCOD2851	236.6
LCOD2852	311.6	LCOD2852	234.8
LCOD2853	314.5	LCOD2853	202.8
LCOD2854	313.4	LCOD2854	240.5
LCOD2855	285.2	LCOD2855	226.4
LCOD2856	307.5	LCOD2856	233.8
LCOD2857	310.3	LCOD2857	242.4
LCOD2858	305.1	LCOD2858	237.7
LCOD2859	309.6	LCOD2859	245
LCOD2860	311.4	LCOD2860	241.7
LCOD2862	271.6	LCOD2862	238.5
LCOD2863	309.9	LCOD2863	225.7
LCOD2864	312.1	LCOD2864	232
LCOD2865	309.8	LCOD2865	231.7
LCOD2866	306.3	LCOD2866	236.4
LCOD2867	301.1	LCOD2867	241.3
LCOD2868	312	LCOD2868	227.5
LCOD2869	309.4	LCOD2869	233.6
LCOD2870	309.3	LCOD2870	231.2
LCOD2871	310.8	LCOD2871	236.3
LCOD2872	308.7	LCOD2872	249.2
LCOD2874	265.3	LCOD2874	242.3
LCOD2875	309.2	LCOD2875	229.8
LCOD2876	305.8	LCOD2876	236.6
rhein	142	LCOD2877	234.8

LCOD2878	279.8	LCOD2878	229
LCOD2879	288	LCOD2879	230.1
LCOD2882	282.4	LCOD2882	236.6
LCOD2883	278.5	LCOD2883	230.1
LCOD2884	276.5	LCOD2884	237.3
LCOD2885	279.2	LCOD2885	237.8
LCOD2886	280.1	LCOD2886	232.2

*Rhein was tested for positive control, and labeled in red.

Table S2. Data collection and refinement statistics[a]

	FTO/MA (4QKN)
**Data collection**	
Space group	H 3
Cell dimensions	
a, *b*, *c* (Å)	141.26, 141.26, 83.58
α, β, γ (°)	90, 90, 120
Resolution (Å)	30.0-2.05 (2.12-2.05)[b]
No. of observations	201804
No. unique	37797 (3228)
R_{sym}[c]	0.068 (0.465)
$I/\sigma(I)$	34.5 (1.6)
Completeness (%)	97.0 (82.8)
Redundancy	5.3 (2.3)
**Data refinement**	
Resolution (Å)	27.86-2.20 (2.27-2.20)
No. reflections	31190 (2774)
$I/\sigma(I)$	36.4 (2.4)
Completeness (%)	98.9 (97.0)
R_{work}/R_{free}	20.2/24.8
No. atoms	
protein	3536
ligand	35
ion	1
water	73
B factors (Å^2)	
protein	48.9
ligand	71.9
Rmsd[d] in	
Bond lengths (Å)	0.010
Bond angles (º)	1.208
Ramachandran Plot[e]	
Favoured (%)	96.0
Allowed (%)	4.0

[a]The structure was solved using one crystal.

[b]Highest resolution shell is shown in parenthesis.

[c]$R_{sym} = \Sigma|(I - \langle I \rangle)|/\Sigma(I)$, where I is the observed intensity.

[d]Root mean squared deviation.

[e]Values calculated in CCP4 suite using Procheck.
